# Metabolic difference between patient-derived xenograft model of pancreatic ductal adenocarcinoma and corresponding primary tumor

**DOI:** 10.1186/s12885-024-12193-x

**Published:** 2024-04-17

**Authors:** Shi Wen, Xianchao Lin, Wei Luo, Yu Pan, Fei Liao, Zhenzhao Wang, Bohan Zhan, Jianghua Feng, Heguang Huang

**Affiliations:** 1 Department of General Surgery, Fujian Medical University Union Hospital, No. 29, Xinquan Road, Gulou District, 351001 Fuzhou, China; 2 Department of Electronic Science, Fujian Provincial Key Laboratory of Plasma and Magnetic Resonance, Xiamen University, No. 422, Siming South Road, Siming District, 361005 Xiamen, China

## Abstract

**Background:**

Patients-derived xenograft (PDX) model have been widely used for tumor biological and pathological studies. However, the metabolic similarity of PDX tumor to the primary cancer (PC) is still unknown.

**Methods:**

In present study, we established PDX model by engrafting primary tumor of pancreatic ductal adenocarcinoma (PDAC), and then compared the tumor metabolomics of PC, the first generation of PDX tumor (PDXG1), and the third generation of PDX tumor (PDXG3) by using ^1^H NMR spectroscopy. Then, we assessed the differences in response to chemotherapy between PDXG1 and PDXG3 and corresponding metabolomic differences in drug-resistant tumor tissues. To evaluate the metabolomic similarity of PDX to PC, we also compared the metabolomic difference of cell-derived xenograft (CDX) vs. PC and PDX vs. PC.

**Results:**

After engraftment, PDXG1 tumor had a low level of lactate, pyruvate, citrate and multiple amino acids (AAs) compared with PC. Metabolite sets enrichment and metabolic pathway analyses implied that glycolysis metabolisms were suppressed in PDXG1 tumor, and tricarboxylic acid cycle (TCA)-associated anaplerosis pathways, such as amino acids metabolisms, were enhanced. Then, after multiple passages of PDX, the altered glycolysis and TCA-associated anaplerosis pathways were partially recovered. Although no significant difference was observed in the response of PDXG1 and PDXG3 to chemotherapy, the difference in glycolysis and amino acids metabolism between PDXG1 and PDXG3 could still be maintained. In addition, the metabolomic difference between PC and CDX models were much larger than that of PDX model and PC, indicating that PDX model still retain more metabolic characteristics of primary tumor which is more suitable for tumor-associated metabolism research.

**Conclusions:**

Compared with primary tumor, PDX models have obvious difference in metabolomic level. These findings can help us design in vivo tumor metabolomics research legitimately and analyze the underlying mechanism of tumor metabolic biology thoughtfully.

**Supplementary Information:**

The online version contains supplementary material available at 10.1186/s12885-024-12193-x.

## Brief summary

The tumor metabolomics of patients-derived xenograft model is different to corresponding primary tumor, which mainly involved glycolysis and TCA-associated anaplerosis pathways of amino acids.

**Supplementary Information:**

The online version contains supplementary material available at 10.1186/s12885-024-12193-x.

## Background

Subcutaneous and orthotopic cell line-derived xenograft (CDX) animal models have been widely used for the thousands of researches and make a great contribution to development of oncological biology and oncological pharmacology [1]. Recently, with the deepening understanding of tumor heterogeneity, scholars realized that CDX models cannot realistically mimic the genetic heterogeneity and biological properties of tumors in human [2–4]. These shortcomings hinder the progress of precision medicine based on five critical elements- clinical bioinformatics, precision methods, disease-specific biomarkers, drug discovery and development, and precision regulations, which are critical for establishing of executable integrative cancer model [1, 5, 6]. There is a general agreement that, although many issues contribute to the current inefficient drug discovery pipelines, deficiencies in in vivo models add substantially to the low rate of success [3]. Thus, to solve this problem, scholars develop animal models which directly graft tissues of patients’ primary tumor into immunodeficient mice, named patient-derived tumor xenograft (PDX) models.

The development of PDX models assumes that the xenograft could faithfully resemble the original tumors, maintaining the molecular features and tumor microenvironment of the primary tumor [7]. Previous reports indicate that PDX models of multiple cancer, including colorectal, breast, bladder, renal and hepatopancreatobiliary cancers, could preserve gene expression pattern, mutational status, drug response and tumor architecture [8–11]. Therefore, PDX models provide a relatively realistic tools for predicting efficacy of treatment and identifying heterogeneous factors for patient-selection strategies [11]. However, recently, several reports indicate that the molecular profiles of PDX models of cancers, like pancreatic cancer and renal carcinoma, are significantly different to their primary tumors, but closely resemble those seen in metastatic and relapsed tumor [12–14]. Cancer cells derived from established PDX tumor models diverged from the primary tumor and their transcriptomic signatures could not be reestablished even regrown in vivo [15]. These findings indicate that host microenvironment could pose an obvious influence on molecular characteristics of PDX, which leaded a differential biological behavior of PDX compared with the primary tumor. In addition, few metabolomic comparison between primary tumor and corresponding PDX tumor established by grafting tumor tissues is reported. Thus, in present study, we compared the tumor metabolomics between primary tumor of pancreatic ductal adenocarcinoma (PDAC) and corresponding PDX models, trying to clarify whether the PDX model could resemble the metabolic signatures of primary tumor of PDAC. Besides, we also evaluate the metabolomic similarity of PDX and CDX model with PC to define the superiority and suitability of PDX model over CDX model.

## Methods

### Ethics statement

The study protocol was approved by the Institutional Review Board of Fujian Medical University Union Hospital (No.27, [2017] FMUUH ethical review, approved in May, 2017)., Fuzhou, China, and was conducted in accordance with the 1964 Declaration of Helsinki and its later amendments or comparable ethical standards. Informed consent was obtained from all patients. All animal experimental protocols were operated in accordance with ARRIVE guidelines and other guidelines like the revised Animals (Scientific Procedures) Act 1986 and the Guide for the Care and Use of Laboratory Animals.

### Primary tumor sample collection

Twenty-seven primary tumor samples were collected from 27 PDAC patients who received PDAC radical surgery in Fujian Medical University Union Hospital from September 2017 to May 2018. After resection, tumors were immediately cleaned up, divided into pieces in size of 0.8 × 0.8 × 0.8 cm and washed with physiological saline. Then, each tumor sample were divided into three parts. One of them was stored in phosphate buffered solution in 4 ℃, prepared for establishment of PDX model. Another one was snap frozen with liquid nitrogen and stored in -80 ℃, used for ^1^H NMR spectroscopy. The last one was disposed with 10% formalin solution for histopathological examination. All tumor sample were confirmed by hematoxylin and eosin (H&E) staining histopathological examination and evaluated independently by two pathologists.

### Cell culture and animal feeding

PDAC cell strain Panc-1(Catalog NO. SCSP-535) were obtained from Shanghai Institute of Cell Biology, Chinese Academy of Sciences (Shanghai, China), At the circumstance of 5% CO2 and 37 °C, Panc-1 was incubated in Dulbecco’s modified eagle medium (DMEM, Gibco, Thermo Fisher Scientific (China) Co., Ltd., Shanghai, China) added with 10% fetal bovine serum (Gibco) in cell incubator (3110, Thermo Scientific). Every 2–3 days, Panc-1 digested by 0.125% trypsinogen (Life Technologies, GrandIsland, NY, USA) for the passage with the ratio of 1:2–4. BALB/c^nu/nu^ mice (male, 4 weeks, weighing 18–20 g), purchased from Shanghai Slac laboratory animals Co., Ltd. (NO: SCXK (HU) 2012-0002), were bred in Fujian Medical University Animals Centre (Fuzhou, china) with a standard SPF-grade laboratory condition.

### Establishment and passage of PDX model

To establish the patient-derived xenograft (PDX) model, a fresh primary sample from each patient was subcutaneously implanted into three Balb/cnu/nu mice (a total of 81 mice) after inducing anesthesia using isoflurane. After a period of 42 days, successful establishment of PDX models was achieved in 34 mice from 13 patients. These mice were divided into two groups: the first generation PDX model (PDXG1) comprising 24 mice, and the first generation PDX model for chemotherapy (PDXG1CH) comprising 10 mice. When the passage tumor volume exceeded 1500 mm^3^ or the diameter of passage tumors reached 1.5 cm, the tumor-bearing mice of PDXG1 were euthanized under anesthesia using isoflurane. The harvested PDX tumors from each patient were divided into three parts. One part underwent triple washing with phosphate-buffered solution and then implanted into new mice for further passages, while the other two parts were collected for ^1^H NMR-based metabolomic analysis and H&E histopathological examination. For tumor passaging to new mice, each fresh tumor samples from PGXG1 were divided into pieces of 0.5*0.5*0.5 cm and remove necrotic tissue. These pieces were subcutaneously implanted into the back of mice by using a metal needle. After two passages of tumor, a total of 28 third generation PDX models were formed from 13 patients (referred to as PDXG3). These PDXG3 models were further divided into two groups: the third generation PDX model (PDXG3) consisting of 18 mice, and the third generation PDX model for chemotherapy (PDXG3CH) consisting of 10 mice. Like PDXG1, the PDXG3 mice were euthanized when the volume of passage tumors exceeded 1500 mm^3^ or the diameter of passage tumors reached 1.5 cm. The tumor samples were also collected for ^1^H NMR-based metabolomic analysis and H&E histopathological examination. The data regarding tumor growth were recorded every three days.

### Albumin bound paclitaxel and gemcitabine treatment

Ten pairs of PDXG1CH and PDXG3CH mice were subjected to intraperitoneal injections of albumin-bound paclitaxel (125mg/m^2^, Abraxane, AbraxisBioscience, LLC) and gemcitabine (100mg/m^2^, Gemzar, Eli Lilly and Company) (AG) every 7 days once the tumor volume reached 50 mm^3^. After four rounds of AG injections, the PDXG1CH and PDXG3CH mice were humanely euthanized, and the harvested tumor tissues were rapidly frozen using liquid nitrogen and fixed with 10% formalin. All tumor growth data were recorded every three days. The pathological response of collected tumors to chemotherapy was evaluated by tumor pathologist based on tumor regression grade (TRG, 8th AJCC).

### Establishment of CDX model

Panc-1 in the exponential phase were digested with 0.125% trypsinogen, washed by phosphate buffer saline (PBS) for three times, then collected and resuspended in PBS (5 × 10^6^/ml). After airway anesthesia, 100 µl of cell suspension was subcutaneously injected into 13 Balb/c^nu/nu^ mice to established cell-derived subcutaneous xenograft (CDSX) models. After 28 days feeding, 13 tumor-bearing mice were sacrificed, and the harvested tumors were divided into pieces in size of 1 × 1 × 1 mm. Then, the space between pancreas and liver of 13 Balb/c^nu/nu^ mice were implanted with tumor pieces by surgeries to establish cell-derived orthotopic xenograft (CDOX) model. After 28 days feeding, 11 CDOX mice had a mercy killing and tumors were collected for ^1^H NMR and histopathological examination.

### Sample preprocessing

All tumor samples (300 mg each) were defrosted on ice. Then, mixed with 0.6 mL ultrapure water and 1.2mL methanol, samples were homogenized for 3 min (MiniBeadbeater-16; BIO SPEC, Bartlesville, OK, USA) in 7-mL lap tubes. After the addition of 1.2 mL chloroform and 1.2 mL ultrapure water, the mixture was vortexed for 60 s. After 15 min standing on ice, each sample was centrifuged at 10,397 g for 10 min and the supernate was collected. Then, the supernate was lyophilized in vacuum freeze-drying equipment (LGJ-10 C; Four-ring Science Instrument Plant, Beijing, China) for 24 h to eliminate water and methanol.

### ^1^H high resolution-NMR spectroscopy

Before ^1^H high resolution-NMR (^1^H HR-NMR) spectroscopy, the lyophilizate was dissolved in 550µL of 150-mM deuterated phosphate buffer (NaH_2_PO_4_ and K_2_HPO_4_, pH 7.4, including 0.1% sodium 3-[trimethylsilyl] propionate-2,2,3,3-d4 [TSP]) followed with a 10-min centrifugation at 10,397 g. Then, the 500µL upper layer liquid was transferred into a 5-mm NMR tube. In present study, NMR spectroscopy was performed on a Bruker AscendTM NMR spectrometer (Bruker Corporation, Karlsruhe, Germany) at 600.13 MHz proton frequency and 295 K. Spectra of all samples were acquired by using the ^1^H NOESYPR1D pulse sequence with water suppression: [RD-90°-t1-90°-tm-90°-ACQ]. A total of 32 scans with a spectral width of 12 kHz, accompanied with a data point of 32 K, were collected for all spectra. The acquisition time was 2.66 s with a relaxation delay (RD) of 4 s, a fixed interval (t1) of 4µs and the mixing time (tm) of 0.1 s. The pulse width of 90° was 11µs.

### Data processing

All free induction decays derived in ^1^H NMR spectroscopy were multiplied by an exponential weighting function equivalent to a line-broadening of 1 Hz to increase the signal-to-noise ratio, followed by a Fourier transformation. Then, all spectra were manually corrected for phase and baseline using MestReNova (V9.0; Mestrelab Research, Santiago de Compostela, Galicia, Spain). The chemical shift in spectra was referenced to TSP at δ0.0. Spectral regions of δ0.5-9.0 were integrally segmented into discrete regions of 0.004ppm. the spectral regions of δ4.61–5.49 and δ3.32–3.39 were removed to eliminate the interference of water and methanol signals for analysis. Then, the integrated data were normalized to 100 prepared for further multivariate and univariate statistical analysis. Resonance assignment and metabolite identification were conducted based on the literature and public databases [16, 17].

### Statistical analysis

In present study, to extract the bioinformation contained in the NMR spectra, multivariate statistical analyses, including principal component analysis (PCA) and orthogonal partial least squares discriminant analysis (OPLS-DA) were conducted to compare the metabolome of tumors between different groups. PCA using mean center scaling was implemented on an online metabolomic database Metaboanalysts 5.0 (https://www.metaboanalyst.ca/, assess on June, 2022). As an unsupervised statistical method, PCA can simplify the multivariate data into a few principal components, which can highlight intrinsic trends, distribution of clusters and the existence of outliers. The OPLS-DA using unit variance scaling was conducted using SIMCA-P+ (Ver.14.0, Umetrics AB, Umea, Sweden) for pairwise comparisons of different groups. As a supervised statistical method, OPLS-DA can maximize the distinction between different groups and establish pattern recognition models for semi-quantitative evaluation of metabolomic difference and discriminatory metabolites identification. For all OPLS-DA, 7-fold cross-validations and response permutation tests were performed to evaluate the fitness and predictability of OPLS-DA models.

### Discriminatory metabolite identification

For screening of discriminatory metabolites, the *Pearson* correlation coefficients (Pcorr) and variable importance in projections (VIP) of metabolites were back calculated based on OPLS-DA models. The cut-off value of Pcorr for each comparison was determined by degree of freedom (df = n-2, *p* < 0.05). To identify discriminatory metabolites more rigorously, the *Student’s t* test was also performed to compare the relative level of metabolites between different groups. The relative levels of metabolites were represented by the integral area under spectral curve of metabolites’ characteristic peaks. The metabolites having *p* value of|Pcorr| and * Student’s t* test less than 0.05 and VIP > 1.00 were identified as discriminatory metabolites. To visualize the discriminatory metabolites for each comparison, color-code volcano plot was drawn by using Matlab (Ver.2021, MathWorks, Natick, MA, US) based on VIP, pcorr, *p* of *t* test and fold change of metabolites.

### Metabolic pathways analysis

For discovering underlying bioinformation, metabolites set enrichment and metabolic pathway analysis were performed by using the Kyoto Encyclopedia of Genes and Genomes (KEGG) [18–20] and MetaboAnalyst 5.0 online databases (https://www.metaboanalyst.ca/, accessed on June, 2022) [21].

## Results

### The metabolomics of the first generation of PDX tumor was different to primary tumor

**Fig. 1:**
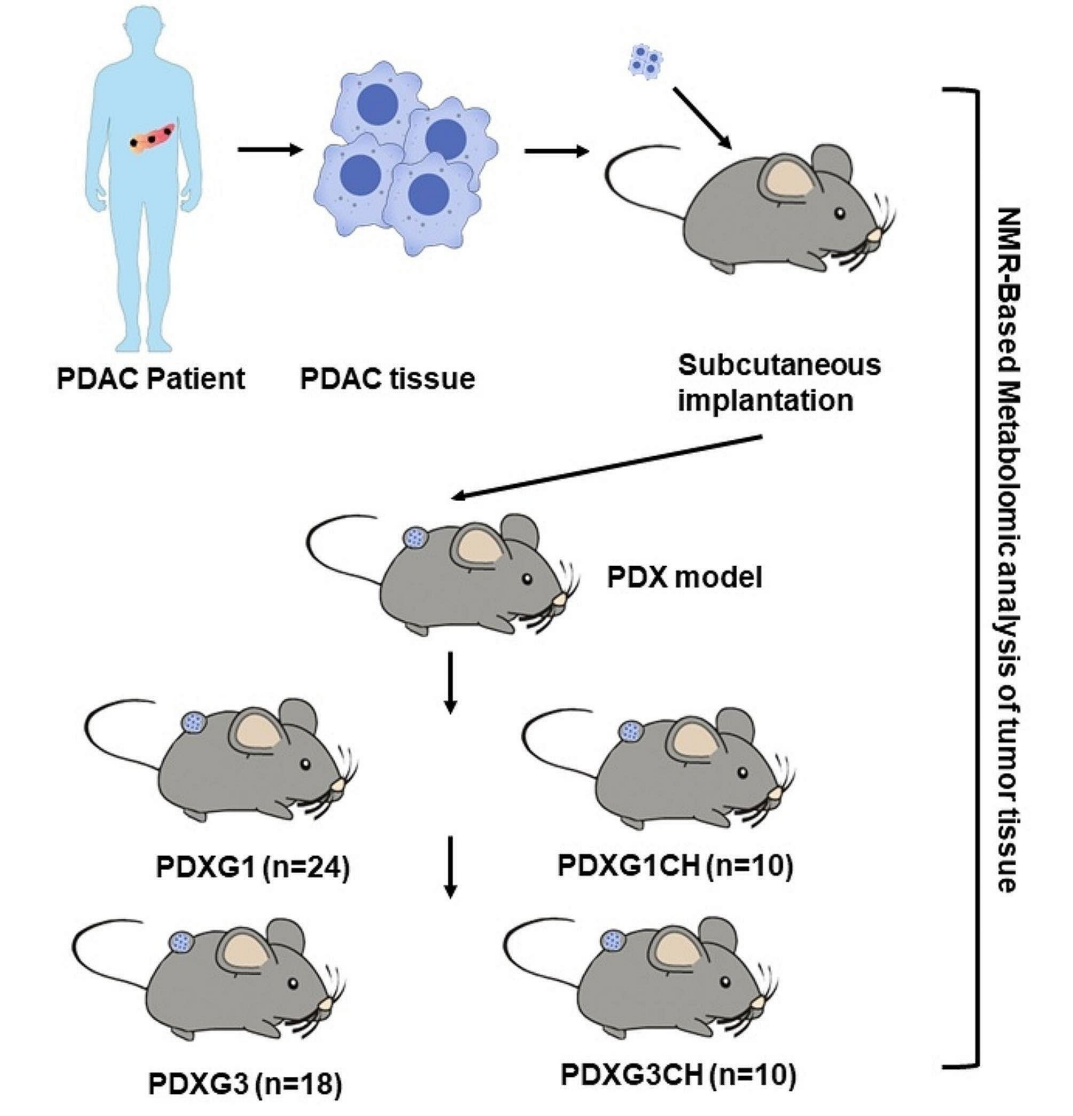
The research process diagram of this study. PDAC, pancreatic ductal adenocarcinoma; PDX, patient-derived xenograft; PDXG1, the first generation of PDX model; PDXG3, the third generation of PDX model; PDXG1CH, the first generation of PDX model receiving chemotherapy; PDXG1CH, the third generation of PDX model receiving chemotherapy

**Fig. 2:**
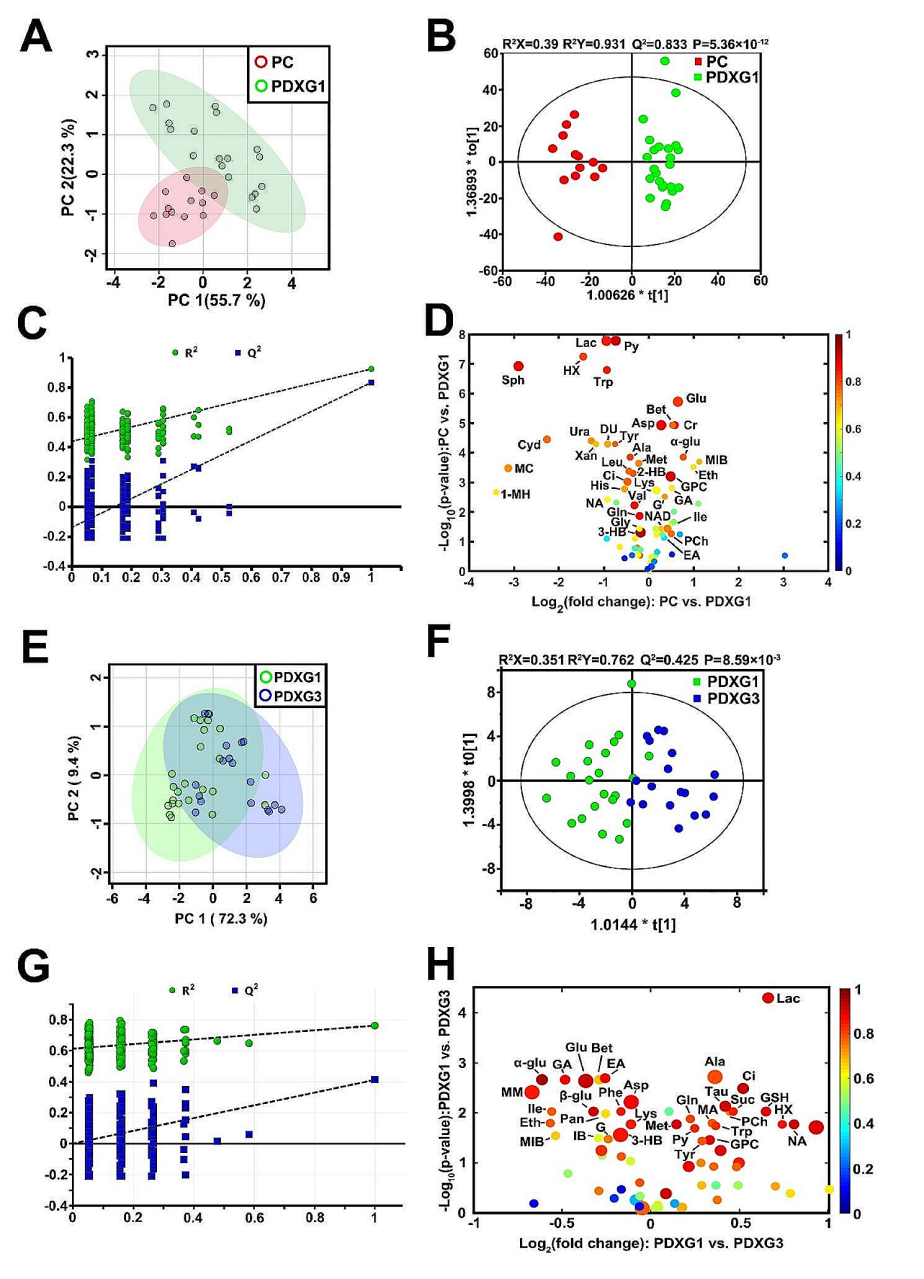
Univariate and multivariate statistical analyses of spectra data derive from tumor of primary cancer (PC), the first generation of patient-derived patients (PDXG1) and the third generation of patient-derived patients (PDXG3). Figure 2A&E. The scores plot of principal component analysis (PCA) of ^1^H NMR spectral data derived from PC vs. PDXG1 (**A**), and from PDXG1 vs. PDXG3 (**E**). Figure 2B&F. The scores plot of orthogonal partial least squares discriminant analysis (OPLS-DA) of ^1^H NMR spectral data derived from PC vs. PDXG1 (**B**), and from PDXG1 vs. PDXG3 (**F**). Figure 2C&G. The response permutation test of OPLS-DA model of PC vs. PDXG1 (**C**) and PDXG1 vs. PDXG3 (**G**). R^2^ and Q^2^ indicate the fitness and predictive performance of the established model. Figure 2D&H. The volcano plots of discriminative metabolites of PC and PDXG1 (**D**), and PDXG1 vs. PDXG3 (**H**). Each dot represents a metabolite. The color of dots represents the absolute value of Pcorr and the size of dot represents the value of VIP. 1-MH: 1-Methylhistidine; DU: 2-Deoxyuridine; 2-HB: 2-Hydroxybutyrate; 3-HB: 3-Hydroxybutyrate; MC: 5-Methylcytidine; Ala: Alanine; Asp: Aspartate; Bet: Betaine; Ci: Citrate; Cr: Creatine; Cyd: Cytidine; Eth: Ethanol; EA: Ethanolamine; Glu: Glutamate; Gln: Glutamine; GSH: Glutathione; G: Glycerol; GPC: Glycerophosphocholine; Gly: Glycine; GA: Guanidoacetate; His: Histidine; HX: Hypoxanthine; IB: Isobutyrate; Ile: Isoleucine; Lac: Lactate; Leu: Leucine; Lys: Lysine; Met: Methionine; MA: Methylamine; MIB: Methyl isobutyrate; MM: Methylmalonate; NA: Nicotinamide; NAD: Nicotinamide adenine dinucleotide; Pan: Pantothenate; PCho: Phosphocholine; Py: Pyruvate; Suc: Succinate; Sph: Sphignosine; Tau: Taurine; Trp: Tryptophan; Tyr: Tyrosine; Ura: Uracil; Val: Valine; Xan: Xanthine; α-glu: α-Glucose; β-glu: β-Glucose

In present study, by subcutaneously implanting tumor slices derived from 13 PDAC primary tumors (PC group), we successfully established 24 first-generation PDX nude mice PDXG1 group) and 18 third-generation PDX mice bearing 18 tumors (PDXG3) (Fig. 1). By using univariate and multivariate statistical analyses, we evaluated the tumor metabolomic difference between PDXG1 model (*n* = 24) and the PC (*n* = 13). As demonstrated in scores plot of PCA, an obvious separation could be seen between the clusters of PC and PDXG1 without outlier exited (Fig. 2A). Then, OPLS-DA was further performed to highlight the specific metabolic difference between PC and PDXG1. The separation of PC and PDX become more obvious in OPLS-DA scores plot (Fig. 2B**).** The parameters of pattern recognition model (R_2_X = 0.39, R_2_Y = 0.931, Q^2^ = 0.833) and the *p*-value of CV-ANOVA (*P* = 5.36 × 10^− 12^) indicated a high stability and reliability of pattern recognition model which was favorable for further screening discriminatory metabolites. The response permutation plot indicated that no overfitting was existed in this model (Fig. 2C).

In present study, a total of 70 metabolites were identified (Table S1). For PC vs. PDXG1, absolute value of Pcorr higher than 0.552 and VIP higher than 1.000 were consider as the cut-off value of discriminatory metabolites. To eliminate the deviation of multivariate statistical analyses, The *p* value of students’ t tests less than 0.05 after false discovery rate (FDR) correction was also set as the cut-off value of discriminatory metabolites distinguishing PC and PDXG1. Compared with PC, 38 discriminatory metabolites were screened out in PDXG1 (Fig. 2D, Table S2). The level of 1-methylhistidine, 2-deoxyuridine, 2-hydroxybutyrate, 3-hydroxybutyrate, 5-methylcytidine, alanine, citrate, cytidine, glutamine, glycine, histidine, hypoxanthine, lactate, leucine, methionine, nicotinamide, pyruvate, sphingosine, tryptophan, tyrosine, valine, xanthine, in PDXG1 tumor were lower. Meanwhile, the level of aspartate, betaine, creatine, ethanol, ethanolamine, glutamate, glycerol, glycerophosphocholine, guanidoacetate, isoleucine, lysine, methylisobutyrate, nicotinamide adenine dinucleotide, phosphocholine, α-glucose in PDXG1 were higher than PC.

### Metabolomic difference was existed during tumor passage of PDX model

To elucidate whether the tumor passage could influence the tumor metabolomics of PDAC PDX model, we compared the tumor metabolomics of first and third generation of PDX model (*n* = 24 and 18, respectively). As scores plot demonstrated, the clusters of PDXG1 and PDXG3 were partially overlapped, indicating the metabolic difference of PDXG1 vs. PDXG3 was relatively slight (Fig. 2E). However, by using OPLS-DA, a pattern recognition model can be established with acceptable model parameters (R^2^X = 0.351, R^2^Y = 0.762, Q^2^ = 0.425 and *p* value of CV-ANOVA = 0.000859), which suggested that a moderate metabolic difference between PDXG1 and PDXG3 (Fig. 2F). The response permutations plot indicated that the model was not overfitted which was suitable for the screening discriminatory metabolites (Fig. 2G).

As demonstrated in Fig. 2H. 33 discriminatory metabolites between PDXG1 and PDXG3 were identified. The level of alanine, citrate, glutamine, glutathione, glycerophosphocholine, hypoxanthine, lactate, methionine, methylamine, nicotinamide, phosphocholine, pyruvate, succinate, taurine, tryptophan, tyrosine, uridine diphosphate glucose in PDXG3 were higher than PDXG1. Meanwhile, the level of 3-hydroxybutyrate, aspartate, betaine, ethanol, ethanolamine, glutamate, glycerol, guanidoacetate, isobutyrate, isoleucine, lysine, methyl isobutyrate, methylmalonate, nicotinamide adenine dinucleotide, pantothenate, phenylalanine, α-glucose, β-glucose in PDXG3 were lower than PDXG1 (Table S3).

### Tricarboxylic acid cycle (TCA)-associated metabolisms were the main metabolic pathways reprogramed during establishment and passage of patient-derived xenograft model

**Fig. 3:**
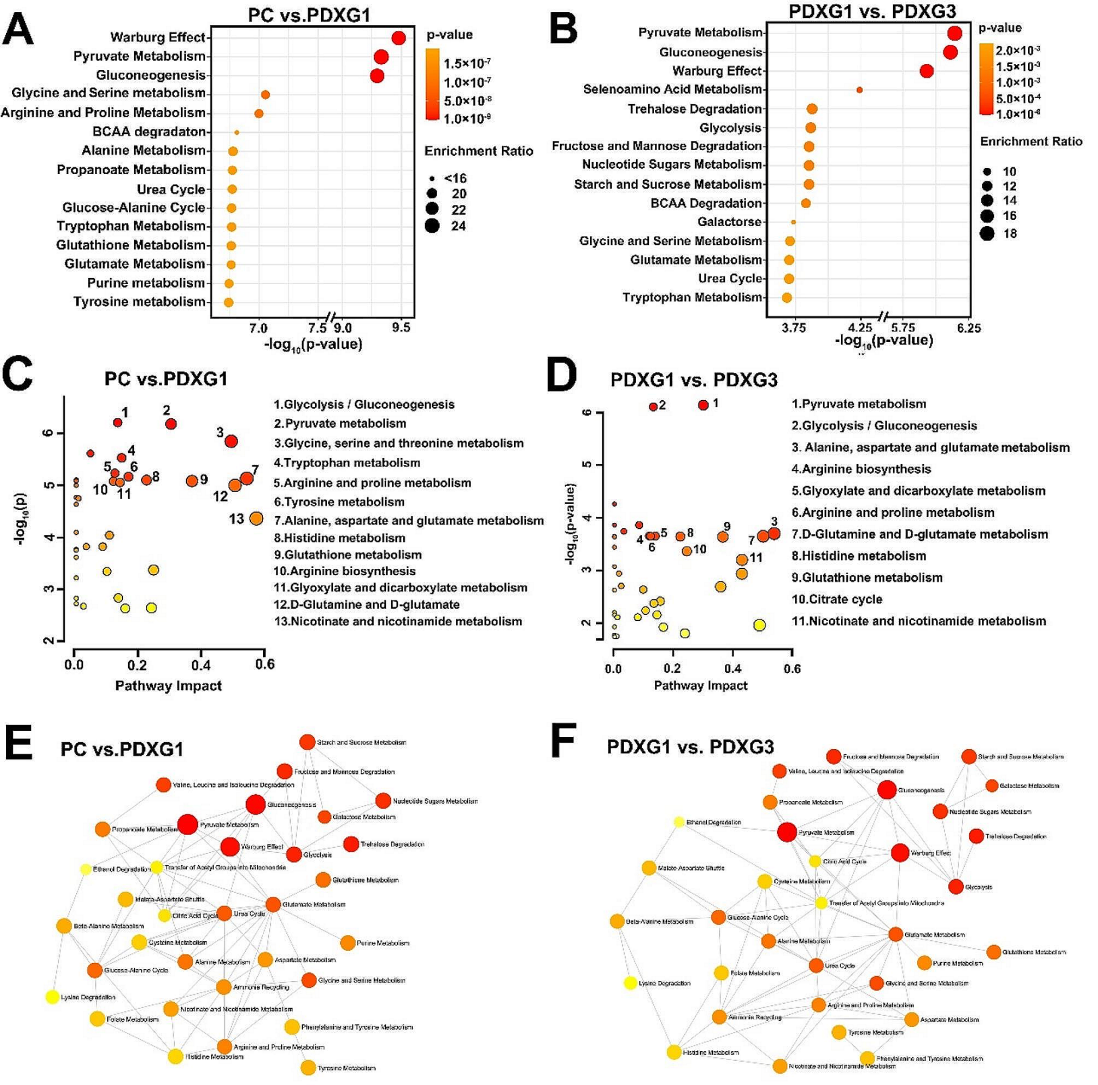
The metabolic enrichment and pathways analyses of PC vs. PDXG1 and PDXG1 vs. PDXG3. Figure 3A&B. The metabolic enrichment analysis based on metabolomic difference of PC vs. PDXG1 and PDXG1 vs. PDXG3, respectively. Y axis represents the top 15 metabolic difference-related metabolic sets. Figure 3C&D. The topological analysis of the metabolic pathway involved in metabolomic difference of PC vs. PDXG1 and PDXG1 vs. PDXG3, respectively. X axis represent the impact of metabolic pathway, and Y axis represent the–log(*p*-value) of pathway. Each dot represents a pathway, and the color of dot represent *p*-value of pathway ranging from low (red) to high (yellow). For PC vs. PDXG1, label dots represented the statistically significant metabolic pathways based on *p*-value less than 1 × 10^− 4^ and pathway impact higher than 0.1. For PDXG1 vs. PDXG3, label dots represented the statistically significant metabolic pathways based on *p*-value less than 1 × 10^− 3^ and pathway impact higher than 0.1. Figure 3E&F. The metabolic network analyses based on metabolomic difference of PC vs. PDXG1 and PDXG1 vs. PDXG3, respectively. Each dot represents a pathway, and the color of dot represent *p*-value of pathway ranging from low (red) to high (yellow). Dashed lines represent significant connection between metabolic pathways

By using relative quantitative metabolites enrichment analysis and metabolic pathway analysis, we found glycolysis/gluconeogenesis, TCA metabolism and TCA-associated metabolic replenishment pathways were highly associated with the metabolomic difference of PC vs. PDXG1 and PDXG1 vs. PDXG3. For the comparison between PC and PDXG1, Warburg effect (aerobic glycolysis), pyruvate metabolism and gluconeogenesis were the major differential metabolic pathways. Besides, multiple amino acids metabolisms like glycine, alanine, aspartate, glutamate, branched-chained amino acids (BCAAs) were also significantly associated with the metabolomic difference between PC and PDXG1 (Fig. 3A&B, Table S4&5). For the comparison between PDXG1 and PDXG3, notably, the pyruvate metabolism, Warburg effect and gluconeogenesis were also the major metabolic pathways associated with the metabolomic difference of PDXG1 vs. PDXG3. Meanwhile, the metabolic pathways of multiple amino acids (AAs), nicotinate and nicotinamide metabolism were significantly associated with the metabolic change in PDXG3 (Figure 3C&D, Table S6&7). Through metabolic network analysis, we noticed that glycolysis and pyruvate metabolisms acted as a core of the metabolic network associated with metabolomic difference of both PC vs. PDXG1 and PDXG1 vs. PDXG3, connecting multiple AAs metabolic pathway (Fig. 3E&F).

**Fig. 4:**
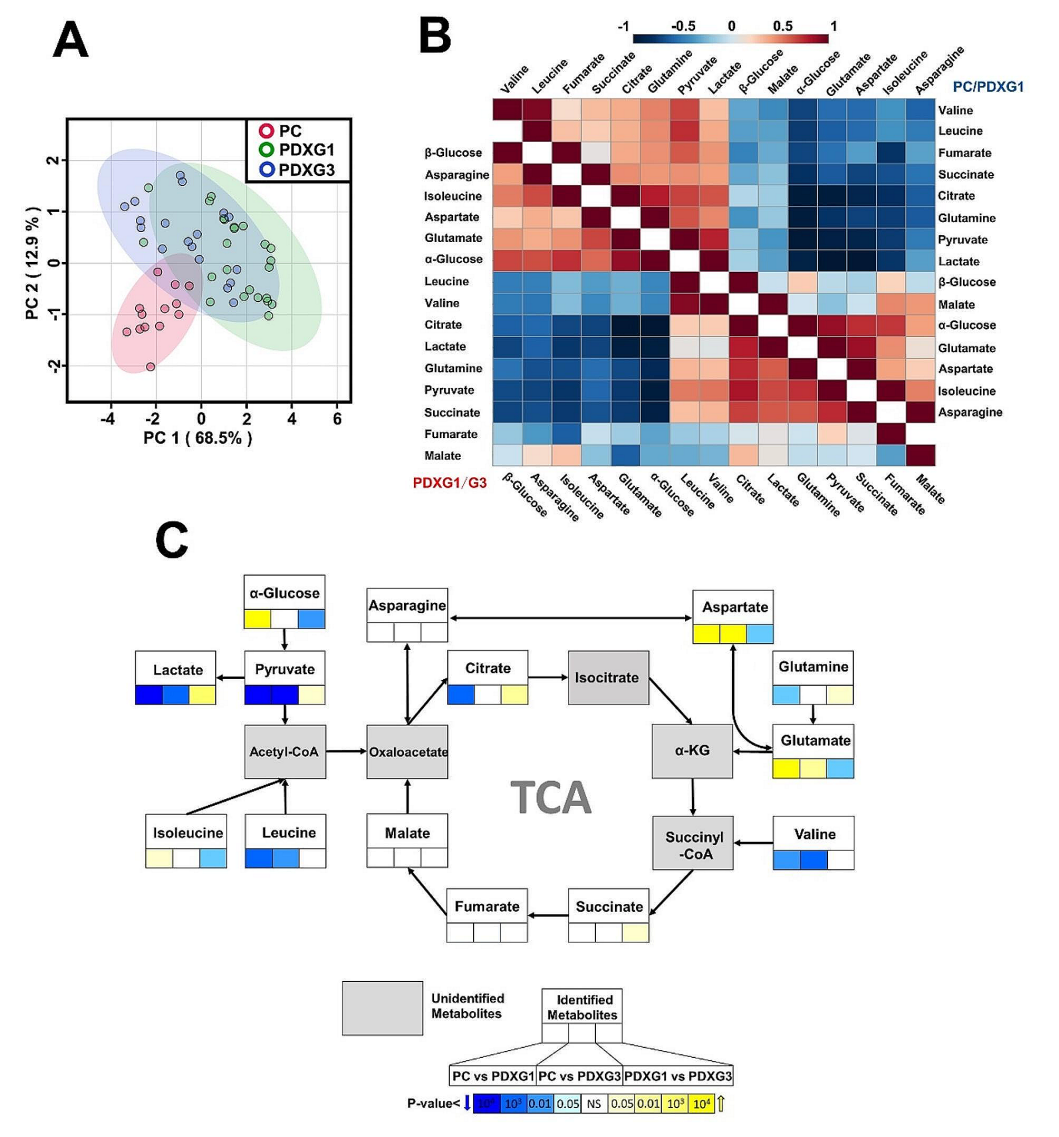
The comprehensive metabolic analysis of metabolomic difference of PC vs. PDXG1 and PDXG1 vs. PDXG3. Figure 4A. The scores plot of principal component analysis (PCA) of ^1^H NMR spectral data derived from PC, PDXG1 and PDXG3. Figure 4B. The heatmap of correlation coefficient between critical metabolites involved in glycolysis/TCA metabolism in comparisons of PC vs. PDXG1 and PDXG1 vs. PDXG3. The cool-toned color of boxes represents a negative correlation while the warm-toned color represents a positive correlation. Figure 4C. The metabolic flux of TCA-associated metabolisms in the metabolomic comparisons between PC, PDXG1 and PDXG3. Each box represents a metabolite. The three solid boxes under metabolites represent the *p* value of t tests of metabolites in comparisons of PC vs. PDXG1, PC vs. PDXG3 and PDXG1 vs. PDXG3, respectively. The cool-toned color of boxes represents that the corresponding metabolite in latter group was significantly lower than the former group, while the warm-toned color represents a relatively high level of metabolites in latter group. KEGG was used as important reference for figure drawing [18–20]

To get insight into metabolomic difference existed in PC vs. PDXG1 and PDXG1 vs. PDXG3, we comprehensively analyzed the metabolomic similarity and difference involved in the comparisons between PC, PDXG1, PDXG3. As demonstrated in scores plot of PCA (Fig. 4A), the cluster of PC was separated with the clusters of PDXG1 and PDXG3 obviously while the clusters of PDXG1 and PDXG3 were heavily overlapped, indicating that human-to-mouse tumor grafting can cause more metabolic reprogramming than mouse-to-mouse tumor passage. The correlation of the critical TCA-associated metabolites in PC vs. PDXG1 and PDXG1 vs. PDXG3 was detailly demonstrated in heatmap plot (Fig. 4B). Interestingly, the levels of lactate and pyruvate were both positively correlated with TCA intermediates, such as citrate, succinate and fumarate, in PC vs. PDXG1 and PDXG1 vs. PDXG3. Meanwhile, the aspartate, glutamate and isoleucine were negative correlated with these TCA intermediates. To visualize the TCA-associated metabolic difference between PC, PDXG1 and PDXG3, a metabolic flux plot was drawn based on relative level of TCA-associated metabolites (Fig. 4C).

### The metabolomic difference between different generations PDX with chemotherapy treated was obvious

**Fig. 5:**
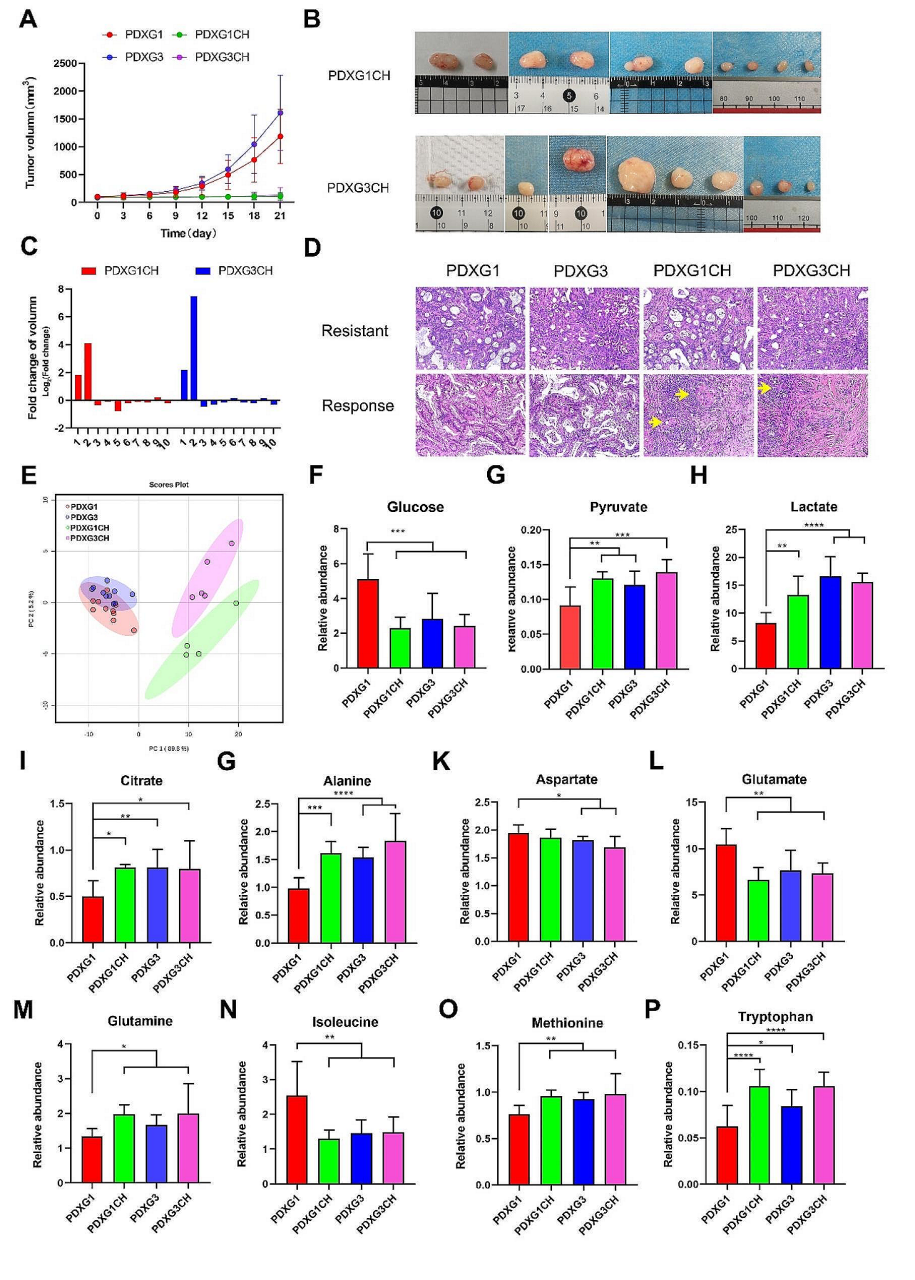
The comparison of growth data and tumor metabolomics between PDXG1, PDXG3, PDXG1CH and PDXG3CH. Figure 5A. The tumor volume line graph of PDXG1, PDXG3, PDXG1CH and PDXG3CH. Figure 5B. The photograph of PDXG1CH and PDXG3CH tumor after receiving Albumin-bound paclitaxel plus gemcitabine (AG) treatment. All mice in PDXG1CH and PDXG3CH had intraperitoneal injections of AG every 7 days and sacrificed after 4 rounds of AG treatment to collect tumor samples. Figure 5C The changes in tumor volume of PDXG1CH and PDXG3CH before and after chemotherapy. Figure 5D The representative H&E pathological images of AG-resistant and AG-response tumor in corresponding PDXG1, PDXG3, PDXG1CH and PDXG3CH groups. Compare with AG-resistant tumor, the AG-response tumor had numerous fibrous stroma and there was significant lymphocyte infiltration after receiving treatment. Meanwhile, only a small amount of residual cancer cells remained (Yellow arrow). Figure 5E. The PCA score plot of metabolomic data of tumor samples from PDXG1, PDXG3, PDXG1CH and PDXG3CH. Figure 5F-P. The histogram of the relative concentration of metabolites associated with glycolysis and amino acids in PDXG1, PDXG1CH, PDXG3 and PDXG3CH.

Since important tumor-related metabolisms, such as TCA, can undergo reprogramming during the establishment and passage of PDX models, it is critical to investigate whether there are differences in the chemosensitivity and post-chemotherapy tumor metabolism among PDX models with different passages. In present study, we treated the first and third generation of PDX models with a combination of albumin-bound paclitaxel and gemcitabine (AG) (PDXG1CH and PDXG3CH group, *n* = 10, respectively). Compared with PDXG1 and PDXG3, AG treatment significantly inhibit the growth of tumor in both PDXG1CH and PDXG3CH group (Fig. 5A). Only 2 of 10 tumors in PDXG1CH and PDXG3CH, respectively, showed increases in tumor volume during AG treatment (Fig. 5B&C). By pathological examination of tumor tissue, 4 of 10 in PDXG1CH and 5 of 10 in PDXG3CH show limited pathological response (TRG ≥ 2) to AG treatment. Meanwhile, 6 of 10 in PDXG1CH and 5 of 10 in PDXG3CH have obviously pathological response(TRG ≤ 1) after AG treatment (Fig. 5D).

Then, we conducted metabolomic profiling on AG-resistant tumor(TRG ≥ 2)from PDXG1CH (*n* = 4) and PDXG3CH (*n* = 5). Interestingly, there are metabolic differences in AG-resistant tumors between the PDXG1CH and PDXG3CH groups. As shown in the PCA score plot (Fig. 5E), principal component 1 mainly represents the metabolic differences formed by AG chemotherapy intervention, while principal component 2 represents the metabolic differences formed by tumor passaging. These results not only demonstrate that the survival pressure induced by chemotherapy exerts a significant shaping effect on tumor metabolism but also suggest that the metabolic differences generated by tumor propagation are still retained in this process. Using univariate statistical analysis, the relative abundances of glycolytic metabolites and amino acids were compared among all groups. Interestingly, key intermediate metabolites of glycolysis such as glucose, lactate, pyruvate, and amino acids showed significant differences in level between the PDXG1 group and the other groups (Fig. 5F-P). However, these substances appeared to have no significant differences among the PDXG3, PDXG1CH, and PDXG3CH groups. These findings suggested that drug-resistant tumors formed after chemotherapy treatment in PDX models with different passage numbers exhibited consistent changes in glycolysis and amino acid metabolism, with greater similarity observed in PDX models with multiple passages.

### PDX model keep metabolic signatures of primary tumor better than CDX model

**Fig. 6:**
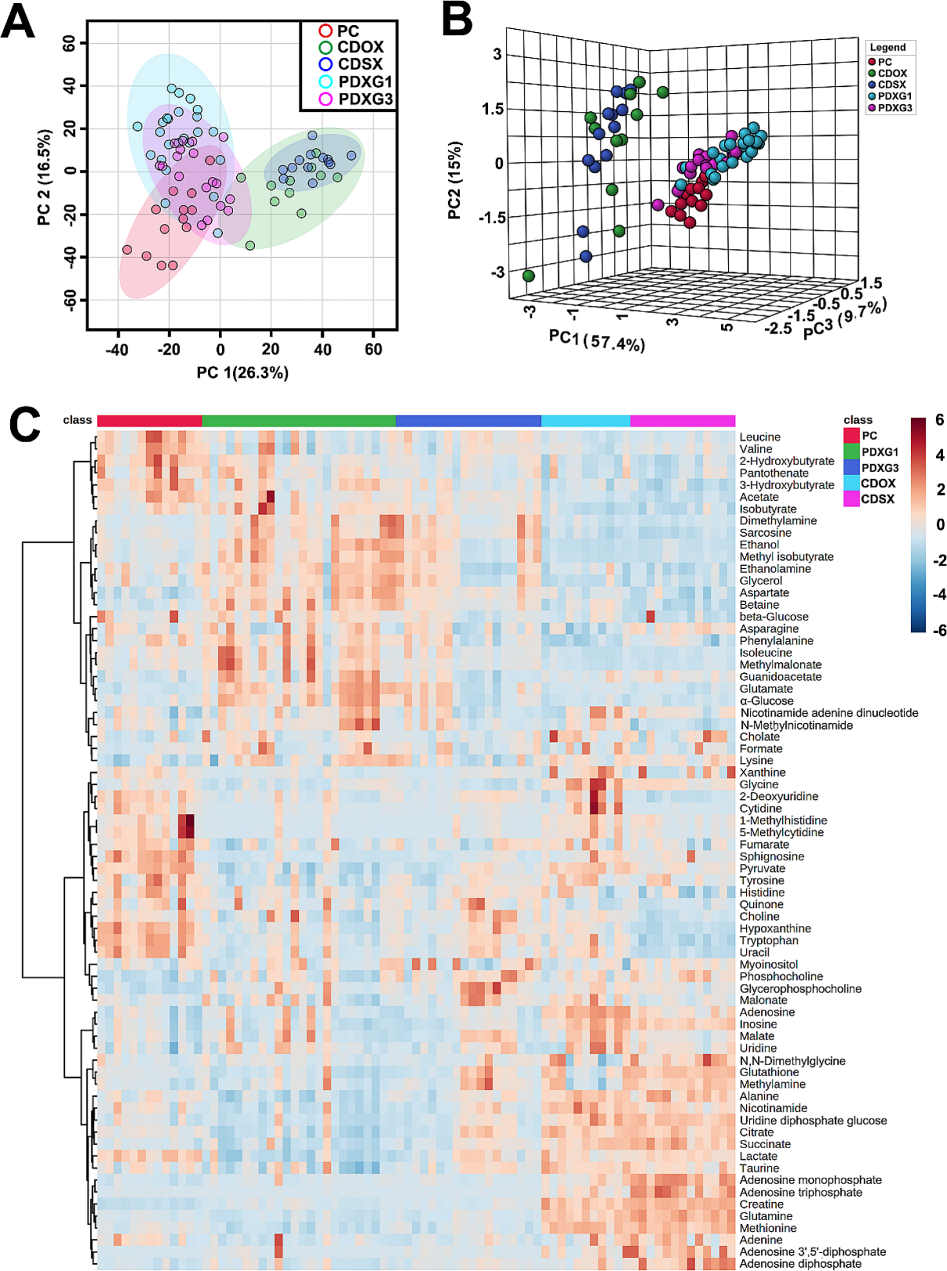
The metabolomic analysis of spectra data derived from tumor of PC, PDX model and CDX models. Figure 6A&B. The 2D and 3D scores plot of principal component analysis (PCA) of ^1^H NMR spectral data derived from PC, PDXG1, PDXG3, CDOX and CDSX. Figure 6C. The heatmap of metabolites’ relative levels in the tumors of PC, PDX model and CDX models. The cool-toned color of boxes represents a relatively low level while the warm-toned color represents a relatively high level of metabolites

To clarify the advantages of PDX models over CDX models in metabolic studies, we compared the ability of PDX and CDX models to mimic the metabolic profile of primary tumors. A total of 13 and 11 subcutaneous and orthotopic xenograft models of Panc-1 cell strain (CDSX, *n* = 13 and CDOX, *n* = 11) were successfully established. As demonstrated on scores plot of PCA, the cluster of PDXG1 and PDXG3 were near to cluster of PC and far from the clusters of CDSX and CDOX (Fig. 6A&B). The heatmap drawn based on relative level of metabolites of all groups illustrated that, compared with CDSX and CDOX, PDXG1 and PDXG3 have more similarity in overall distribution of metabolites levels compared with PC (Fig. 6C).

## Discussion

Tumor animal models are critical platforms for investigating the biological features of tumor, discovering genesis and progress mechanism and screening underlying anticancer agents. With the development of PDX models, scholars and physicians gain access to an new experimental tool carrying tumor heterogeneity, which empowers people to evaluate and treat tumor in individual [22]. One of the advantages of PDX model is a well gene mutation preservation of primary tumor. However, for stroma-rich tumor like PDAC, engraftment and passages of tumor could impose significant influence on expression of genes associated with stroma compartment and inflammation. Mattie et al. reported that the PDX model established by grafting PDAC tumor tissue demonstrated significantly expression change of metastatic gene signatures which may contribute to successful establishment of PDX models [12]. Garrido-Laguna et al. reported that PDX tumors were more often SMAD4 mutant and had a metastatic gene expression signature [14]. Being the downstream of genomics, metabolomics inevitably reflected the genetic reprogramming of PDX model during the grafting of tumor [23]. However, by far, no research has been conducted to assess the metabolic maintenance of PDX model compared with the primary tumor.

In present study, we firstly compared the metabolomics of tumor derived from the first generation of PDX with the primary tumor of patients and found the levels of glycolysis-related and TCA-related metabolites like lactate, pyruvate, and citrate in PDXG1 were significantly lower than the primary tumors. This finding strongly suggested that bioenergy metabolism of tumor had an obvious reprogramming after engraftment from patients to mice, and the glycolysis in PDXG1 was suppressed, leading to a shortage of pyruvate and its downstream metabolites. For pancreatic cancer, aerobic glycolysis (Warburg’s effect) was one of the most critical metabolic reprogramming during genesis and development of neoplasia [24]. In PDAC, tumors contain oxygenated and hypoxic regions, so normoxic and hypoxic cancer cells are coexisted. The hypoxic cancer cells mainly depend on glycolysis to produce energy and secrete lactate to promote formation of acidosis microenvironment [25]. The lactate generated by hypoxic cancer cells can get into the circulation and then be utilized by normoxic PDAC cells to feed TCA. Through this metabolic pathway, circulating lactate of PDAC patients is increased and provides over two-fold of TCA substrates (like citrate and fumarate) than glucose [26]. Thus, the lower level of lactate and pyruvate in PDXG1 model suggested, compared with primary tumor, the ability of initial PDX model to generate endogenous lactate and utilize circulating lactate was weakened, partly explaining why the success rate of PDX model is low.

Interestingly, we found the decrease of pyruvate and glycolysis in PDXG1 did not be accompanied with a significant decrease in the flux of TCA cycle. Although the levels of citrate and pyruvate in PDXG1 was decreased compared with PC, the other intermediates of TCA like succinate, malate and fumarate did not decrease, indicating that substrates of TCA were replenished by anaplerosis pathways. Glutaminolysis is one of the critical anaplerosis pathways to compensate for the shortage of TCA cycle substrates due to the limited pyruvate availability caused by enhanced glycolysis in cancer cells [27]. Glutamine is the most abundant circulating amino acid in blood and muscle. During glutaminolysis, glutamine was converted to glutamate catalyzed by glutaminase 1/2, and then converted to α-ketoglutarate to fuel TCA. However, as previously reported, PDAC with oncogenic KRAS relies on a distinct pathway in which glutamine-derived aspartate is transported into the cytoplasm where it can be converted into oxaloacetate by aspartate transaminase [28]. Then, this oxaloacetate can be converted into malate and pyruvate to fuel TCA. Recently reported, this KRAS-regulated glutamine anaplerosis pathways requires mitochondrial uncoupling protein 2-mediated aspartate transportation [29]. Inhibiting this glutamine metabolism could heavily hamper the growth of PDAC cells. Like glutamate, asparagine is also a critical non-essential AA for the growth of PDAC. In absence of exogeneous glutamine, cancer cells can sustain glutamate-dependent process through *de novo* glutamate biosynthesis, with exception of asparagine [30]. Inability to sustain cellular asparagine limit the growth of glutamine-restricted cancers. Thus, in present study, PDXG1 had lower levels of glutamine and pyruvate, and higher levels of glutamate and aspartate than PC, indicating that, after engraftment, glutamine/aspartate-based anaplerosis of TCA may be enhanced in PDXG1 models to sustain TCA flux.

Another potential anaplerosis pathway of TCA was the degradation of BCAA in PDXG1. For PDAC, branched-chain amino acid transaminase (BCAT)-mediated BCAAs catabolism plays a critical role in development and progress of tumor. In cancer cells, BCAAs are converted to branched-chain α-keto acids (BCKAs) including α-ketoisocaproate, α-keto-β-methylvalerate, and α-ketoisovalerate catalyzed by BCATs. These BCAKs are then converted to acetyl-CoA and succinyl-CoA to replenish TCA flux. As previously reported, BCAA can enhance growth of PDAC in a dose-dependent manner. Inhibiting BCAT2-mediated BCAA catabolism ameliorates formation of precancerous lesions of pancreas [31]. Moreover, the degradation of BCAT2 in PDAC cells is promoted by acetylation of lysine 44 residue and enhance growth of PDAC [32]. For stromal-rich PDAC, cancer-associated fibroblasts (CAFs) provide BCKAs through BCAT1-mediated BCAA catabolism to fuel cancer cells [33]. Thus, as found in present study, the levels of leucine and valine were significant decreased in PDXG1 compared with PC, implying an enhanced catabolism of BCAA to replenish the flux of TCA.

To clarify the metabolic alteration accompanied with the passages of PDX models, we compared the tumor metabolomics between the first and the third generation of PDX model, finding that glycolysis and pyruvate metabolisms were still the main pathways associated with metabolomic difference. Compared with PDXG1, the level of glycolysis-associated metabolites like lactate and pyruvate were significantly increased in PDXG3, indicating that, during passages of PDX model, the aerobic glycolysis can be partially recovered from a suppressed status. Meanwhile, the level of glutamine in tumor of PDXG3 was increased and glutamate is decreased, also implying that the level glutaminolysis was partially recovered from an enhanced status in PDXG1. Besides, the levels of valine and leucine in PDXG3 were not different to that in PDXG3, suggested that the enhanced metabolic replenishment of BCAA degradation toward TCA can be maintained during passages of PDX models. These metabolic reprogramming may be attributed to the replacement of human-derived stroma components (vessels) by mouse-derived stroma during the process of establishment and passages of PDX models. Meanwhile, the subsets of cancer cells which adapted to mouse microenvironment rather than human microenvironment gradually become the main population companied with corresponding metabolic reprogramming. These processes are accompanied with the emerge of specific clonal selection and protumorigenic signatures [34–36], which often occur in metastatic tumor [12–14, 37, 38]. At this point, it is unclear whether the mouse can only support and provide a unique protumorigenic environment. Besides, although the tumor response of different generation of PDX to AG treatment was not different, the metabolomic difference still retained. For most studies on anti-tumor drugs, including metabolomics, glycolysis and amino acid metabolism remain core pathways of research. The metabolic differences formed during the establishment and passage of patient-derived xenograft (PDX) models can still have potential implications on the interpretation of experimental results and the determination of targets. However, the global metabolomic changes sometimes cannot reflect the specific changes in metabolic activities of cancer cells, which may be interfered by stromal cells. Space metabolomics based on AFADESI-MSI may help to solve this problem. Therefore, how to utilize PDX model for metabolism-associated studies still needs to be further evaluated.

In this study, we also found that the metabolomic similarity between PDX-derived and primary tumors are obviously better than CDX-derived tumor, either subcutaneous or orthotopic xenograft. For CDX models, each cell strain used for establishing model is derived from only one patient’s tumor and is replicated in culture medium before grafting into mice, which mean the unique metabolic reprogramming in specific cell strain can be very different to common metabolic changes in PDAC cancer. In addition, tumors of CDX models were formed by proliferation of human PDAC cell added with the infiltration of mice-derived cells like lymphocytes and fibroblasts, which mean CDX models lack human-derived stroma and human-derived infiltrating cells. These factors may significantly hamper CDX models to mimic metabolic activity of human cancer. Meanwhile, long-term passages of cell lines in vitro could also lead to a loss or alteration of tumor-related genes. These factors jointly determine metabolomics of CDX models would have significantly metabolic difference to primary tumor, which can be far larger than PDX model. However, these findings still require comprehensive research to further elucidate.

However, due to limitation of NMR-based metabolomic method, only 70 metabolites were identified in this study. Many metabolites included in analysis are intermediates of amino acids, glycolysis and TCA cycle metabolisms, which may potentially lead to overestimation of related pathways in enrichment analysis. The relatively small sample size and identified metabolites inevitably limited deeper discoveries, and in the future, a metabolomics dataset containing more sample sizes and metabolites may help improve accuracy of research analysis and provide richer information for PDX modeling and passage-related metabolomics change.

## Conclusion

The present study demonstrated that PDX tumor metabolomics was obviously differ to that of primary tumor. Compared to primary tumor, the tumor of PDX models have a lower level of glycolysis and an enhanced TCA-associated anaplerosis metabolism. These metabolomic reprogramming of initial PDX model were partly recovered during the passages of PDX model. The metabolic difference due to passages of PDX model can be retained after

AG-treatment. However, PDX model can mimic the metabolic environment of primary tumor better than CDX models. These findings can help us design in vivo tumor metabolomics research legitimately and analyze the underlying mechanism of tumor metabolic biology thoughtfully.

### Electronic supplementary material

Below is the link to the electronic supplementary material.

Supplementary Material 1

Supplementary Material 2

Supplementary Material 3

Supplementary Material 4

Supplementary Material 5

Supplementary Material 6

Supplementary Material 7

## Data Availability

The integral data of ^1^H NMR spectra is available from corresponding author on reasonable request.

## Abbreviations

AA: Amino acid
BCKA: Branched-chain α-keto acid
BCAA: Branched-chained amino acid
CDX: Cell-derived xenograft
H&E: Hematoxylin and eosin
CDOX: Orthotopic cell-derived xenograft
PDAC: Pancreatic ductal adenocarcinoma
PDX: Patients-derived xenograft
Pcorr: Pearson correlation coefficients
PC: Primary cancer
TSP: Sodium 3-[trimethylsilyl] propionate-2,2,3,3-d4
CSDX: Subcutaneous cell-derived xenograft
PDXG1: The first generation of PDX tumor
PDXG3: The third generation of PDX tumor
TCA: Tricarboxylic acid cycle
VIP: Variable importance in projections
