# Supplementary material for: Metabolic difference between patient-derived xenograft model of pancreatic ductal adenocarcinoma and corresponding primary tumor
Source: BMC Cancer. 2024 Apr 17;24:485. doi: 10.1186/s12885-024-12193-x (PMC11022326; doi:10.1186/s12885-024-12193-x)
Supplement: Supplementary file 1 — Supplementary Material 1 [file 12885_2024_12193_MOESM1_ESM.docx]

| Table S1. Assignment of metabolites base on ^1^H NMR spectra of tumor from PC, PDX and CDX models | | | |
| --- | --- | --- | --- |
| Number | Metabolites | abbrevation | Chemical shift^*^ |
| 1 | 1-Methylhistidine | 1-MH | 7.69s |
| 2 | 2-Deoxyuridine | DU | 5.95d;7.87d |
| 3 | 2-Hydroxybutyrate | 2-HB | 0.90t;1.70m |
| 4 | 3-Hydroxybutyrate | 3-HB | 1.20d;2.31dd;4.16m |
| 5 | 5-Methylcytidine | MC | 7.69d |
| 6 | Acetate | Ace | 1.92s |
| 7 | Adenine | Ad | 8.11s;8.12s |
| 8 | Adenosine | Ade | 4.29dd;4.44dd;6.11d;8.37s |
| 9 | Adenosine 3',5'-diphosphate | PAP | 8.59s |
| 10 | Adenosine diphosphate | ADP | 8.55s |
| 11 | Adenosine monophosphate | AMP | 4.52d;6.15d;8.27s;8.61s |
| 12 | Adenosine triphosphate | ATP | 8.53s |
| 13 | Alanine | Ala | 1.48d |
| 14 | Asparagine | Asn | 2.88d;2.95dd;3.99dd |
| 15 | Aspartate | Asp | 2.70dd;2.81dd;3.94dd |
| 16 | Betaine | Bet | 3.91s |
| 17 | Cholate | CL | 0.72m |
| 18 | Choline | Cho | 3.21s |
| 19 | Citrate | Ci | 2.53d;2.67d |
| 20 | Creatine | Cr | 3.04d;3.93s |
| 21 | Cytidine | Cyd | 5.87d;7.81d |
| 22 | Dimethylamine | DMA | 2.76s |
| 23 | Ethanol | Eth | 1.19t |
| 24 | Ethanolamine | EA | 3.15t;3.86t |
| 25 | Formate | For | 8.46s |
| 26 | Fumarate | Fum | 6.52s |
| 27 | Glutamate | Glu | 2.07m;2.1m;2.35m;3.78t |
| 28 | Glutamine | Gln | 2.14m;2.45m |
| 29 | Glutathione | GSH | 2.56m;2.99m |
| 30 | Glycerol | G | 3.56dd;3.66dd |
| 31 | Glycerophosphocholine | GPC | 3.24s;3.68m;4.33m |
| 32 | Glycine | Gly | 3.57s |
| 33 | Guanidoacetate | GA | 3.8s |
| 34 | Histidine | His | 7.09s;7.93s |
| 35 | Hypoxanthine | HX | 8.2s;8.22s |
| 36 | Inosine | Ino | 4.26dd;6.08;8.24s;8.34s |
| 37 | Isobutyrate | IB | 1.07d |
| 38 | Isoleucine | Ile | 0.94t;1.01d;1.26m |
| 39 | Lactate | Lac | 1.33d;4.11d |
| 40 | Leucine | Leu | 0.96t;1.7m |
| 41 | Lysine | Lys | 1.48m;1.73m;1.92m;3.03m;3.76m |
| 42 | Malate | Mal | 4.31dd |
| 43 | Malonate | M | 3.12s |
| 44 | Methionine | Met | 2.14s;2.65t |
| 45 | Methyl isobutyrate | MIB | 1.18d |
| 46 | Methylamine | MA | 2.60s |
| 47 | Methylmalonate | MM | 1.22d |
| 48 | Myo-inositol | MI | 3.29t;3.54dd;3.62t;4.07t |
| 49 | N,N-Dimethylglycine | DMG | 2.92s |
| 50 | Nicotinamide | NA | 7.60dd;8.72dd;8.94d |
| 51 | Nicotinamide adenine dinucleotide | NAD | 4.37m;4.42m;4.49m;4.51m;6.04d;6.09d;6.12d;8.14d;8.43s;8.83d; |
| 52 | N-Methylnicotinamide | MNA | 4.49s;8.91d;8.98d; |
| 53 | Pantothenate | Pan | 0.87s;0.91s |
| 54 | Phenylalanine | Phe | 4.00m;7.33d;7.38t;7.43m |
| 55 | Phosphocholine | PC | 3.22s;4.18m |
| 56 | Pyruvate | Py | 2.39s |
| 57 | Quinone | Qu | 6.81s |
| 58 | Sarcosine | Sar | 2.74s |
| 59 | Sphignosine | Sph | 5.52dd;5.56dd;5.74dd |
| 60 | Succinate | Suc | 2.41s |
| 61 | Taurine | Tau | 3.27t;3.43t |
| 62 | Tryptophan | Trp | 7.28m;7.53d;7.74d |
| 63 | Tyrosine | Tyr | 6.91d;7.20d |
| 64 | Uracil | Ura | 5.80d;7.55d |
| 65 | Uridine | Ud | 4.24t;4.36t;5.91d;5.93d;7.89d |
| 66 | Uridine diphosphate glucose | UDG | 5.61dd;5.97m;7.96d |
| 67 | Valine | Val | 0.99d;1.05d;2.28m |
| 68 | Xanthine | Xan | 7.91s |
| 69 | α-Glucose | α-Glc | 3.74m;3.84m; |
| 70 | β-Glucose | β-Glc | 3.47dd; |

^*^ : s, single peak; d, double peak; dd: dual double peak; t, triple peak; m, multiple; br, broad peak
