# Supplementary material for: Metabolic difference between patient-derived xenograft model of pancreatic ductal adenocarcinoma and corresponding primary tumor
Source: BMC Cancer. 2024 Apr 17;24:485. doi: 10.1186/s12885-024-12193-x (PMC11022326; doi:10.1186/s12885-024-12193-x)
Supplement: Supplementary file 2 — Supplementary Material 2 [file 12885_2024_12193_MOESM2_ESM.docx]

| Table S2. The tumor discriminatory metabolites between PC and PDXG1 | | | | |
| --- | --- | --- | --- | --- |
| Metabolites | PC vs. PDXG1 | | | |
|  | Pcorr^1^ | Fold change^2^ | P^3^ | VIP^4^ |
| 1-Methylhistidine | -0.642 | 0.095 | 2.167×10^-3^ | 1.443 |
| 2-Deoxyuridine | -0.710 | 0.530 | 5.092×10^-5^ | 1.645 |
| 2-Hydroxybutyrate | -0.791 | 0.785 | 4.985×10^-4^ | 1.582 |
| 3-Hydroxybutyrate | -0.948 | 0.882 | 4.906×10^-2^ | 1.953 |
| 5-Methylcytidine | -0.730 | 0.114 | 3.327×10^-4^ | 1.698 |
| Alanine | -0.814 | 0.751 | 1.412×10^-4^ | 1.605 |
| Aspartate | 0.884 | 1.213 | 1.173×10^-5^ | 1.901 |
| Betaine | 0.769 | 1.445 | 1.173×10^-5^ | 1.588 |
| Citrate | -0.782 | 0.721 | 9.526×10^-4^ | 1.772 |
| Creatine | 0.868 | 1.497 | 1.173×10^-5^ | 1.845 |
| Cytidine | -0.767 | 0.208 | 3.562×10^-5^ | 1.799 |
| Ethanol | 0.647 | 1.993 | 3.055×10^-4^ | 1.383 |
| Ethanolamine | 0.565 | 1.111 | 3.680×10^-2^ | 1.111 |
| Glutamate | 0.818 | 1.570 | 1.894×10^-6^ | 1.877 |
| Glutamine | -0.879 | 0.868 | 1.356×10^-2^ | 1.757 |
| Glycerol | 0.721 | 1.281 | 3.052×10^-3^ | 1.462 |
| Glycerophosphocholine | 0.907 | 1.403 | 6.262×10^-4^ | 1.906 |
| Glycine | -0.623 | 0.868 | 3.680×10^-2^ | 1.344 |
| Guanidoacetate | 0.635 | 1.427 | 1.530×10^-3^ | 1.230 |
| Histidine | -0.708 | 0.689 | 1.673×10^-3^ | 1.634 |
| Hypoxanthine | -0.811 | 0.364 | 5.684×10^-8^ | 1.810 |
| Isoleucine | 0.565 | 1.424 | 2.384×10^-2^ | 1.483 |
| Lactate | -0.849 | 0.520 | 1.676×10^-8^ | 1.868 |
| Leucine | -0.771 | 0.736 | 4.194×10^-4^ | 1.571 |
| Lysine | 0.626 | 1.126 | 1.864×10^-3^ | 1.736 |
| Methionine | -0.744 | 0.857 | 2.281×10^-4^ | 1.621 |
| Methyl isobutyrate | 0.692 | 2.183 | 2.021×10^-4^ | 1.478 |
| Nicotinamide | -0.620 | 0.530 | 3.794×10^-3^ | 1.385 |
| NAD | 0.676 | 1.211 | 3.657×10^-2^ | 1.361 |
| Phosphocholine | 0.747 | 1.335 | 3.657×10^-2^ | 1.748 |
| Pyruvate | -0.920 | 0.601 | 1.654×10^-8^ | 1.863 |
| Sphignosine | -0.880 | 0.134 | 1.199×10^-7^ | 1.870 |
| Tryptophan | -0.848 | 0.524 | 1.616×10^-7^ | 1.831 |
| Tyrosine | -0.777 | 0.595 | 5.092×10^-5^ | 1.519 |
| Uracil | -0.729 | 0.412 | 3.966×10^-5^ | 1.567 |
| Valine | -0.867 | 0.802 | 5.935×10^-3^ | 1.714 |
| Xanthine | -0.682 | 0.444 | 5.092×10^-5^ | 1.528 |
| α-Glucose | 0.791 | 1.704 | 1.406×10^-4^ | 1.644 |

^1^ Pcorr: correlation coefficient, positive and negative signs indicate positive and negative corre-lation in the concentrations, respectively. The correlation coefficients of |Pcorr| > 0.552 were used as the cutoff value for the statistical significance. ^2^ The concentration ratio between PC and PDXG1. 3 The p value of *Student’s* t test. The p−values less than 0.05 were used as the cutoff value for the statistical significance. ^4^ Variable importance in projection. More than 1 was the cutoff value of VIP for the statistical significance. NAD：Nicotinamide adenine dinucleotide.
