# Supplementary material for: Metabolic difference between patient-derived xenograft model of pancreatic ductal adenocarcinoma and corresponding primary tumor
Source: BMC Cancer. 2024 Apr 17;24:485. doi: 10.1186/s12885-024-12193-x (PMC11022326; doi:10.1186/s12885-024-12193-x)
Supplement: Supplementary file 3 — Supplementary Material 3 [file 12885_2024_12193_MOESM3_ESM.docx]

| Table S3. The tumor discriminatory metabolites between PDXG1 and PDXG3 | | | | |
| --- | --- | --- | --- | --- |
| Metabolites | PDXG1 vs. PDXG3 | | | |
|  | Pcorr^1^ | Fold change^2^ | P^3^ | VIP^4^ |
| 3-Hydroxybutyrate | -0.853 | 0.889 | 2.752×10^-2^ | 1.824 |
| Alanine | 0.798 | 1.286 | 1.907×10^-3^ | 1.862 |
| Aspartate | -0.876 | 0.927 | 6.057×10^-3^ | 1.899 |
| Betaine | -0.679 | 0.818 | 2.164×10^-3^ | 1.649 |
| Citrate | 0.923 | 1.435 | 3.240×10^-3^ | 1.764 |
| Ethanol | -0.789 | 0.675 | 1.600×10^-2^ | 1.457 |
| Ethanolamine | -0.882 | 0.837 | 2.022×10^-3^ | 1.673 |
| Glutamate | -0.924 | 0.776 | 2.296×10^-3^ | 1.842 |
| Glutamine | 0.810 | 1.168 | 1.316×10^-2^ | 1.472 |
| Glutathione | 0.893 | 1.570 | 9.373×10^-3^ | 1.633 |
| Glycerol | -0.758 | 0.847 | 3.336×10^-2^ | 1.209 |
| Glycerophosphocholine | 0.901 | 1.260 | 3.480×10^-2^ | 1.671 |
| Guanidoacetate | -0.872 | 0.716 | 2.164×10^-3^ | 1.746 |
| Hypoxanthine | 0.851 | 1.673 | 1.690×10^-2^ | 1.576 |
| Isobutyrate | -0.594 | 0.816 | 3.179×10^-2^ | 1.508 |
| Isoleucine | -0.782 | 0.679 | 9.373×10^-3^ | 1.495 |
| Lactate | 0.892 | 1.583 | 5.011×10^-5^ | 1.790 |
| Lysine | -0.846 | 0.926 | 1.690×10^-2^ | 1.639 |
| Methionine | 0.910 | 1.106 | 1.690×10^-2^ | 1.705 |
| Methyl isobutyrate | -0.672 | 0.690 | 2.841×10^-2^ | 1.455 |
| Methylamine | 0.788 | 1.272 | 1.600×10^-2^ | 1.412 |
| Methylmalonate | -0.848 | 0.629 | 3.855×10^-3^ | 1.903 |
| Nicotinamide | 0.914 | 1.752 | 1.690×10^-2^ | 1.651 |
| Pantothenate | -0.651 | 0.839 | 1.035×10^-2^ | 1.551 |
| Phenylalanine | -0.853 | 0.890 | 9.373×10^-3^ | 1.583 |
| Phosphocholine | 0.746 | 1.364 | 9.373×10^-3^ | 1.495 |
| Pyruvate | 0.839 | 1.188 | 2.030×10^-2^ | 1.327 |
| Succinate | 0.847 | 1.381 | 9.373×10^-3^ | 1.438 |
| Taurine | 0.914 | 1.339 | 7.351×10^-3^ | 1.770 |
| Tryptophan | 0.827 | 1.290 | 1.812×10^-2^ | 1.589 |
| Tyrosine | 0.791 | 1.222 | 3.634×10^-2^ | 1.225 |
| α-Glucose | -0.953 | 0.654 | 2.164×10^-3^ | 1.752 |
| β-Glucose | -0.912 | 0.800 | 9.373×10^-3^ | 1.745 |

^1^ Pcorr: correlation coefficient, positive and negative signs indicate positive and negative corre-lation in the concentrations, respectively. The correlation coefficients of |Pcorr| > 0.468 were used as the cutoff value for the statistical significance. ^2^ The concentration ratio between PDXG1 and PDXG3. ^3^ The p value of *Student’s* t test. The p−values less than 0.05 were used as the cutoff value for the statistical significance. ^4^ Variable importance in projection. More than 1 was the cutoff value of VIP for the statistical significance.
