# Supplementary material for: Metabolic difference between patient-derived xenograft model of pancreatic ductal adenocarcinoma and corresponding primary tumor
Source: BMC Cancer. 2024 Apr 17;24:485. doi: 10.1186/s12885-024-12193-x (PMC11022326; doi:10.1186/s12885-024-12193-x)
Supplement: Supplementary file 4 — Supplementary Material 4 [file 12885_2024_12193_MOESM4_ESM.docx]

| **Table S4 The statistical result of enrichment analysis of PC vs PDXG1** | | | | | | | |
| --- | --- | --- | --- | --- | --- | --- | --- |
| **Metabolite Set** | **Total** | **Hits** | **Statistic** | **Expected** | **P value^1^** | **Holm P^2^** | **FDR^3^** |
| Warburg Effect | 58 | 11 | 64.951 | 2.7778 | 3.33E-10 | 3.06E-08 | 1.55E-08 |
| Pyruvate Metabolism | 48 | 9 | 67.286 | 2.7778 | 4.67E-10 | 4.25E-08 | 1.55E-08 |
| Gluconeogenesis | 35 | 7 | 65.072 | 2.7778 | 5.06E-10 | 4.56E-08 | 1.55E-08 |
| Glycine and Serine Metabolism | 59 | 14 | 53.244 | 2.7778 | 8.83E-08 | 7.86E-06 | 6.70E-07 |
| Arginine and Proline Metabolism | 53 | 11 | 53.399 | 2.7778 | 1.00E-07 | 8.81E-06 | 6.70E-07 |
| Valine, Leucine and Isoleucine Degradation | 60 | 9 | 50.452 | 2.7778 | 1.54E-07 | 1.34E-05 | 6.70E-07 |
| Alanine Metabolism | 17 | 7 | 54.189 | 2.7778 | 1.66E-07 | 1.43E-05 | 6.70E-07 |
| Propanoate Metabolism | 42 | 8 | 53.455 | 2.7778 | 1.68E-07 | 1.43E-05 | 6.70E-07 |
| Urea Cycle | 29 | 10 | 53.645 | 2.7778 | 1.68E-07 | 1.43E-05 | 6.70E-07 |
| Glucose-Alanine Cycle | 13 | 4 | 53.987 | 2.7778 | 1.70E-07 | 1.43E-05 | 6.70E-07 |
| Tryptophan Metabolism | 60 | 6 | 53.985 | 2.7778 | 1.70E-07 | 1.43E-05 | 6.70E-07 |
| Glutathione Metabolism | 21 | 6 | 54.017 | 2.7778 | 1.72E-07 | 1.43E-05 | 6.70E-07 |
| Glutamate Metabolism | 49 | 12 | 53.344 | 2.7778 | 1.72E-07 | 1.43E-05 | 6.70E-07 |
| Purine Metabolism | 74 | 14 | 53.466 | 2.7778 | 1.79E-07 | 1.43E-05 | 6.70E-07 |
| Tyrosine Metabolism | 72 | 6 | 54.115 | 2.7778 | 1.81E-07 | 1.43E-05 | 6.70E-07 |
| Beta-Alanine Metabolism | 34 | 6 | 54.056 | 2.7778 | 1.81E-07 | 1.43E-05 | 6.70E-07 |
| Malate-Aspartate Shuttle | 10 | 3 | 54.124 | 2.7778 | 1.82E-07 | 1.43E-05 | 6.70E-07 |
| Ammonia Recycling | 32 | 11 | 53.444 | 2.7778 | 1.82E-07 | 1.43E-05 | 6.70E-07 |
| Aspartate Metabolism | 35 | 8 | 53.758 | 2.7778 | 1.82E-07 | 1.43E-05 | 6.70E-07 |
| Phenylalanine and Tyrosine Metabolism | 28 | 6 | 54.261 | 2.7778 | 1.83E-07 | 1.43E-05 | 6.70E-07 |
| Cysteine Metabolism | 26 | 6 | 54.146 | 2.7778 | 1.85E-07 | 1.43E-05 | 6.70E-07 |
| Histidine Metabolism | 43 | 7 | 54.13 | 2.7778 | 1.85E-07 | 1.43E-05 | 6.70E-07 |
| Amino Sugar Metabolism | 33 | 6 | 54.133 | 2.7778 | 1.86E-07 | 1.43E-05 | 6.70E-07 |
| Folate Metabolism | 29 | 5 | 54.171 | 2.7778 | 1.86E-07 | 1.43E-05 | 6.70E-07 |
| Nicotinate and Nicotinamide Metabolism | 37 | 8 | 53.859 | 2.7778 | 1.89E-07 | 1.43E-05 | 6.70E-07 |
| Arachidonic Acid Metabolism | 69 | 2 | 54.295 | 2.7778 | 1.89E-07 | 1.43E-05 | 6.70E-07 |
| Retinol Metabolism | 37 | 3 | 37.147 | 2.7778 | 7.73E-07 | 5.10E-05 | 2.63E-06 |
| Lysine Degradation | 30 | 3 | 42.513 | 2.7778 | 2.72E-06 | 1.77E-04 | 8.95E-06 |
| Catecholamine Biosynthesis | 20 | 1 | 43.214 | 2.7778 | 9.86E-06 | 6.31E-04 | 3.02E-05 |
| Thyroid hormone synthesis | 13 | 1 | 43.214 | 2.7778 | 9.86E-06 | 6.31E-04 | 3.02E-05 |
| Fructose and Mannose Degradation | 32 | 4 | 38.762 | 2.7778 | 3.31E-05 | 0.0020529 | 9.11E-05 |
| Nucleotide Sugars Metabolism | 20 | 5 | 38.646 | 2.7778 | 3.35E-05 | 0.0020529 | 9.11E-05 |
| Starch and Sucrose Metabolism | 31 | 5 | 38.646 | 2.7778 | 3.35E-05 | 0.0020529 | 9.11E-05 |
| Glycolysis | 25 | 6 | 37.788 | 2.7778 | 3.43E-05 | 0.0020529 | 9.11E-05 |
| Trehalose Degradation | 11 | 4 | 38.258 | 2.7778 | 3.47E-05 | 0.0020529 | 9.11E-05 |
| Galactose Metabolism | 38 | 7 | 32.898 | 2.7778 | 3.93E-05 | 0.0022386 | 1.00E-04 |
| Selenoamino Acid Metabolism | 28 | 6 | 24.947 | 2.7778 | 5.45E-05 | 0.0030493 | 1.35E-04 |
| Spermidine and Spermine Biosynthesis | 18 | 2 | 37.081 | 2.7778 | 6.33E-05 | 0.003484 | 1.53E-04 |
| Glycerolipid Metabolism | 25 | 5 | 22.274 | 2.7778 | 4.05E-04 | 0.021852 | 9.55E-04 |
| Methylhistidine Metabolism | 4 | 1 | 28.595 | 2.7778 | 6.51E-04 | 0.034477 | 0.001496 |
| Carnitine Synthesis | 22 | 4 | 27.853 | 2.7778 | 7.28E-04 | 0.037842 | 0.001633 |
| Biotin Metabolism | 8 | 2 | 28.042 | 2.7778 | 7.51E-04 | 0.038288 | 0.001645 |
| Transfer of Acetyl Groups into Mitochondria | 22 | 6 | 19.313 | 2.7778 | 0.0010465 | 0.052324 | 0.002239 |
| Citric Acid Cycle | 32 | 8 | 18.71 | 2.7778 | 0.0010953 | 0.053671 | 0.00229 |
| Ethanol Degradation | 19 | 5 | 18.068 | 2.7778 | 0.0012687 | 0.060895 | 0.002594 |
| Pyrimidine Metabolism | 59 | 8 | 18.489 | 2.7778 | 0.0017509 | 0.082293 | 0.003502 |
| Methionine Metabolism | 43 | 10 | 13.764 | 2.7778 | 0.0048487 | 0.22304 | 0.009491 |
| Phenylacetate Metabolism | 9 | 3 | 18.496 | 2.7778 | 0.0057035 | 0.25666 | 0.010932 |
| Betaine Metabolism | 21 | 7 | 13.741 | 2.7778 | 0.0063749 | 0.28049 | 0.011969 |
| Porphyrin Metabolism | 40 | 2 | 14.359 | 2.7778 | 0.0099036 | 0.42586 | 0.018223 |
| Vitamin K Metabolism | 14 | 1 | 16.964 | 2.7778 | 0.011313 | 0.47515 | 0.020408 |
| Phospholipid Biosynthesis | 29 | 4 | 13.073 | 2.7778 | 0.016203 | 0.66431 | 0.025434 |
| Fatty acid Metabolism | 43 | 3 | 14.266 | 2.7778 | 0.017815 | 0.7126 | 0.025434 |
| Mitochondrial Beta-Oxidation of Short Chain Saturated Fatty Acids | 27 | 3 | 14.266 | 2.7778 | 0.017815 | 0.7126 | 0.025434 |
| Mitochondrial Beta-Oxidation of Medium Chain Saturated Fatty Acids | 27 | 3 | 14.266 | 2.7778 | 0.017815 | 0.7126 | 0.025434 |
| Mitochondrial Beta-Oxidation of Long Chain Saturated Fatty Acids | 28 | 3 | 14.266 | 2.7778 | 0.017815 | 0.7126 | 0.025434 |
| Butyrate Metabolism | 19 | 4 | 13.646 | 2.7778 | 0.017857 | 0.7126 | 0.025434 |
| Phosphatidylcholine Biosynthesis | 14 | 5 | 13 | 2.7778 | 0.01906 | 0.7126 | 0.025434 |
| Sphingolipid Metabolism | 40 | 5 | 14.455 | 2.7778 | 0.019499 | 0.7126 | 0.025434 |
| Pterine Biosynthesis | 29 | 2 | 14.426 | 2.7778 | 0.020348 | 0.7126 | 0.025434 |
| Androgen and Estrogen Metabolism | 33 | 2 | 14.426 | 2.7778 | 0.020348 | 0.7126 | 0.025434 |
| Androstenedione Metabolism | 24 | 2 | 14.426 | 2.7778 | 0.020348 | 0.7126 | 0.025434 |
| Threonine and 2-Oxobutanoate Degradation | 20 | 3 | 14.423 | 2.7778 | 0.020392 | 0.7126 | 0.025434 |
| Glycerol Phosphate Shuttle | 11 | 2 | 14.425 | 2.7778 | 0.020394 | 0.7126 | 0.025434 |
| Mitochondrial Electron Transport Chain | 19 | 5 | 13.75 | 2.7778 | 0.020395 | 0.7126 | 0.025434 |
| Caffeine Metabolism | 24 | 1 | 14.429 | 2.7778 | 0.020397 | 0.7126 | 0.025434 |
| Fatty Acid Elongation In Mitochondria | 35 | 1 | 14.429 | 2.7778 | 0.020397 | 0.7126 | 0.025434 |
| Steroidogenesis | 43 | 1 | 14.429 | 2.7778 | 0.020397 | 0.7126 | 0.025434 |
| Plasmalogen Synthesis | 26 | 1 | 14.429 | 2.7778 | 0.020397 | 0.7126 | 0.025434 |
| De Novo Triacylglycerol Biosynthesis | 9 | 1 | 14.429 | 2.7778 | 0.020397 | 0.7126 | 0.025434 |
| Cardiolipin Biosynthesis | 11 | 1 | 14.429 | 2.7778 | 0.020397 | 0.7126 | 0.025434 |
| Estrone Metabolism | 24 | 1 | 14.429 | 2.7778 | 0.020397 | 0.7126 | 0.025434 |
| Phytanic Acid Peroxisomal Oxidation | 26 | 4 | 13.744 | 2.7778 | 0.020452 | 0.7126 | 0.025434 |
| Ketone Body Metabolism | 13 | 2 | 13.75 | 2.7778 | 0.020458 | 0.7126 | 0.025434 |
| Fatty Acid Biosynthesis | 35 | 2 | 11.254 | 2.7778 | 0.026377 | 0.7126 | 0.032355 |
| Riboflavin Metabolism | 20 | 4 | 12.216 | 2.7778 | 0.033386 | 0.7126 | 0.039428 |
| Pentose Phosphate Pathway | 29 | 3 | 12.252 | 2.7778 | 0.033428 | 0.7126 | 0.039428 |
| Thiamine Metabolism | 9 | 3 | 12.252 | 2.7778 | 0.033428 | 0.7126 | 0.039428 |
| Steroid Biosynthesis | 48 | 3 | 9.1898 | 2.7778 | 0.037085 | 0.7126 | 0.043188 |
| Bile Acid Biosynthesis | 65 | 6 | 10.275 | 2.7778 | 0.05144 | 0.7126 | 0.059155 |
| Taurine and Hypotaurine Metabolism | 12 | 1 | 10.185 | 2.7778 | 0.054189 | 0.7126 | 0.061548 |
| Pantothenate and CoA Biosynthesis | 21 | 4 | 8.0936 | 2.7778 | 0.063014 | 0.7126 | 0.070699 |
| Phosphatidylethanolamine Biosynthesis | 12 | 4 | 8.1937 | 2.7778 | 0.071352 | 0.71352 | 0.079089 |
| Pyruvaldehyde Degradation | 10 | 2 | 7.3826 | 2.7778 | 0.098721 | 0.88849 | 0.10812 |
| Lactose Synthesis | 20 | 3 | 7.2127 | 2.7778 | 0.10776 | 0.88849 | 0.11663 |
| Sulfate/Sulfite Metabolism | 22 | 2 | 4.2521 | 2.7778 | 0.21604 | 1 | 0.22586 |
| Beta Oxidation of Very Long Chain Fatty Acids | 17 | 2 | 4.2521 | 2.7778 | 0.21604 | 1 | 0.22586 |
| Lactose Degradation | 9 | 2 | 4.2521 | 2.7778 | 0.21604 | 1 | 0.22586 |
| Oxidation of Branched Chain Fatty Acids | 26 | 3 | 3.7204 | 2.7778 | 0.25224 | 1 | 0.26074 |
| Inositol Metabolism | 33 | 4 | 1.7427 | 2.7778 | 0.46375 | 1 | 0.46885 |
| Inositol Phosphate Metabolism | 26 | 4 | 1.7427 | 2.7778 | 0.46375 | 1 | 0.46885 |
| Phosphatidylinositol Phosphate Metabolism | 17 | 3 | 0.1458 | 2.7778 | 0.8226 | 1 | 0.8226 |

^1^ P value of t’ test; ^2^ the P value of t’ test with Holm’ adjustment; ^3^ False discover rate.
