# Supplementary material for: Metabolic difference between patient-derived xenograft model of pancreatic ductal adenocarcinoma and corresponding primary tumor
Source: BMC Cancer. 2024 Apr 17;24:485. doi: 10.1186/s12885-024-12193-x (PMC11022326; doi:10.1186/s12885-024-12193-x)
Supplement: Supplementary file 5 — Supplementary Material 5 [file 12885_2024_12193_MOESM5_ESM.docx]

| **Table S5 The statistical result of enrichment analysis of PDXG1 vs PDXG3** | | | | | | | |
| --- | --- | --- | --- | --- | --- | --- | --- |
| **Metabolite Set** | **Total** | **Hits** | **Statistic** | **Expected** | **P value^1^** | **Holm P^2^** | **FDR^3^** |
| Pyruvate Metabolism | 48 | 9 | 47.804 | 2.5641 | 7.19E-07 | 6.61E-05 | 3.56E-05 |
| Gluconeogenesis | 35 | 7 | 45.643 | 2.5641 | 7.74E-07 | 7.05E-05 | 3.56E-05 |
| Warburg Effect | 58 | 11 | 43.372 | 2.5641 | 1.17E-06 | 1.06E-04 | 3.60E-05 |
| Selenoamino Acid Metabolism | 28 | 6 | 24.029 | 2.5641 | 5.74E-05 | 0.0051102 | 6.40E-04 |
| Trehalose Degradation | 11 | 4 | 32.007 | 2.5641 | 1.33E-04 | 0.011676 | 6.40E-04 |
| Glycolysis | 25 | 6 | 31.762 | 2.5641 | 1.36E-04 | 0.011823 | 6.40E-04 |
| Fructose and Mannose Degradation | 32 | 4 | 31.891 | 2.5641 | 1.40E-04 | 0.012031 | 6.40E-04 |
| Nucleotide Sugars Metabolism | 20 | 5 | 31.845 | 2.5641 | 1.40E-04 | 0.012031 | 6.40E-04 |
| Starch and Sucrose Metabolism | 31 | 5 | 31.845 | 2.5641 | 1.40E-04 | 0.012031 | 6.40E-04 |
| Valine, Leucine and Isoleucine Degradation | 60 | 9 | 29.017 | 2.5641 | 1.48E-04 | 0.012271 | 6.40E-04 |
| Galactose Metabolism | 38 | 7 | 23.446 | 2.5641 | 1.84E-04 | 0.01511 | 6.40E-04 |
| Glycine and Serine Metabolism | 59 | 14 | 29.365 | 2.5641 | 1.96E-04 | 0.015855 | 6.40E-04 |
| Glutamate Metabolism | 49 | 12 | 30.035 | 2.5641 | 1.99E-04 | 0.015895 | 6.40E-04 |
| Urea Cycle | 29 | 10 | 30.153 | 2.5641 | 1.99E-04 | 0.015895 | 6.40E-04 |
| Tryptophan Metabolism | 60 | 6 | 30.311 | 2.5641 | 2.07E-04 | 0.016112 | 6.40E-04 |
| Glucose-Alanine Cycle | 13 | 4 | 30.312 | 2.5641 | 2.07E-04 | 0.016112 | 6.40E-04 |
| Glutathione Metabolism | 21 | 6 | 30.301 | 2.5641 | 2.08E-04 | 0.016112 | 6.40E-04 |
| Alanine Metabolism | 17 | 7 | 30.355 | 2.5641 | 2.08E-04 | 0.016112 | 6.40E-04 |
| Propanoate Metabolism | 42 | 8 | 29.953 | 2.5641 | 2.15E-04 | 0.016112 | 6.40E-04 |
| Arginine and Proline Metabolism | 53 | 11 | 29.291 | 2.5641 | 2.19E-04 | 0.016112 | 6.40E-04 |
| Purine Metabolism | 74 | 14 | 29.882 | 2.5641 | 2.20E-04 | 0.016112 | 6.40E-04 |
| Ammonia Recycling | 32 | 11 | 29.847 | 2.5641 | 2.21E-04 | 0.016112 | 6.40E-04 |
| Aspartate Metabolism | 35 | 8 | 29.948 | 2.5641 | 2.21E-04 | 0.016112 | 6.40E-04 |
| Nicotinate and Nicotinamide Metabolism | 37 | 7 | 30.104 | 2.5641 | 2.22E-04 | 0.016112 | 6.40E-04 |
| Amino Sugar Metabolism | 33 | 6 | 30.185 | 2.5641 | 2.23E-04 | 0.016112 | 6.40E-04 |
| Beta-Alanine Metabolism | 34 | 6 | 30.218 | 2.5641 | 2.25E-04 | 0.016112 | 6.40E-04 |
| Tyrosine Metabolism | 72 | 6 | 30.221 | 2.5641 | 2.25E-04 | 0.016112 | 6.40E-04 |
| Malate-Aspartate Shuttle | 10 | 3 | 30.228 | 2.5641 | 2.26E-04 | 0.016112 | 6.40E-04 |
| Phenylalanine and Tyrosine Metabolism | 28 | 6 | 30.281 | 2.5641 | 2.26E-04 | 0.016112 | 6.40E-04 |
| Folate Metabolism | 29 | 5 | 30.246 | 2.5641 | 2.28E-04 | 0.016112 | 6.40E-04 |
| Cysteine Metabolism | 26 | 6 | 30.224 | 2.5641 | 2.28E-04 | 0.016112 | 6.40E-04 |
| Histidine Metabolism | 43 | 7 | 30.224 | 2.5641 | 2.28E-04 | 0.016112 | 6.40E-04 |
| Arachidonic Acid Metabolism | 69 | 2 | 30.277 | 2.5641 | 2.29E-04 | 0.016112 | 6.40E-04 |
| Citric Acid Cycle | 32 | 8 | 20.966 | 2.5641 | 3.77E-04 | 0.022254 | 0.0010203 |
| Retinol Metabolism | 37 | 3 | 24.708 | 2.5641 | 3.88E-04 | 0.022512 | 0.0010203 |
| Transfer of Acetyl Groups into Mitochondria | 22 | 6 | 20.891 | 2.5641 | 4.22E-04 | 0.02407 | 0.0010791 |
| Vitamin K Metabolism | 14 | 1 | 27.63 | 2.5641 | 4.96E-04 | 0.027753 | 0.0012323 |
| Lysine Degradation | 30 | 3 | 24.943 | 2.5641 | 5.45E-04 | 0.029972 | 0.0013193 |
| Bile Acid Biosynthesis | 65 | 6 | 24.362 | 2.5641 | 0.0011114 | 0.060014 | 0.0026217 |
| Taurine and Hypotaurine Metabolism | 12 | 1 | 24.535 | 2.5641 | 0.0011552 | 0.061226 | 0.002657 |
| Oxidation of Branched Chain Fatty Acids | 26 | 3 | 22.691 | 2.5641 | 0.0018454 | 0.095961 | 0.0041178 |
| Ethanol Degradation | 19 | 5 | 16.093 | 2.5641 | 0.0019006 | 0.09693 | 0.0041178 |
| Phospholipid Biosynthesis | 29 | 4 | 20.284 | 2.5641 | 0.0019246 | 0.09693 | 0.0041178 |
| Phosphatidylcholine Biosynthesis | 14 | 5 | 20.352 | 2.5641 | 0.0020433 | 0.10012 | 0.0042724 |
| Pyruvaldehyde Degradation | 10 | 2 | 22.13 | 2.5641 | 0.0021386 | 0.10265 | 0.0043723 |
| Sphingolipid Metabolism | 40 | 5 | 21.723 | 2.5641 | 0.0023739 | 0.11157 | 0.0047479 |
| Glycerolipid Metabolism | 25 | 5 | 15.56 | 2.5641 | 0.0036825 | 0.16939 | 0.0071487 |
| Pyrimidine Metabolism | 59 | 8 | 16.325 | 2.5641 | 0.0038459 | 0.17307 | 0.0071487 |
| Porphyrin Metabolism | 40 | 2 | 16.931 | 2.5641 | 0.0038585 | 0.17307 | 0.0071487 |
| Phenylacetate Metabolism | 9 | 3 | 18.98 | 2.5641 | 0.0038852 | 0.17307 | 0.0071487 |

^1^ P value of t’ test; ^2^ the P value of t’ test with Holm’ adjustment; ^3^ False discover rate.
