# Supplementary material for: Metabolic difference between patient-derived xenograft model of pancreatic ductal adenocarcinoma and corresponding primary tumor
Source: BMC Cancer. 2024 Apr 17;24:485. doi: 10.1186/s12885-024-12193-x (PMC11022326; doi:10.1186/s12885-024-12193-x)
Supplement: Supplementary file 6 — Supplementary Material 6 [file 12885_2024_12193_MOESM6_ESM.docx]

| **Table S6 The statistical result of metabolic pathway analysis of PC vs PDXG1** | | | | | |
| --- | --- | --- | --- | --- | --- |
| Pathway Name | p^1^ | -log(p) | Holm p^2^ | FDR^3^ | Impact |
| Glycolysis / Gluconeogenesis | 6.16E-07 | 6.2107 | 2.95E-05 | 1.57E-05 | 0.13055 |
| Pyruvate metabolism | 6.56E-07 | 6.1833 | 3.08E-05 | 1.57E-05 | 0.29859 |
| Glycine, serine and threonine metabolism | 1.41E-06 | 5.8502 | 6.49E-05 | 2.26E-05 | 0.48778 |
| Sphingolipid metabolism | 2.42E-06 | 5.6164 | 1.09E-04 | 2.83E-05 | 0.04462 |
| Tryptophan metabolism | 2.94E-06 | 5.5311 | 1.30E-04 | 2.83E-05 | 0.14305 |
| Arginine and proline metabolism | 5.81E-06 | 5.2356 | 2.50E-04 | 2.99E-05 | 0.12158 |
| Tyrosine metabolism | 6.78E-06 | 5.1687 | 2.85E-04 | 2.99E-05 | 0.16435 |
| Alanine, aspartate and glutamate metabolism | 7.32E-06 | 5.1355 | 3.00E-04 | 2.99E-05 | 0.53686 |
| Porphyrin and chlorophyll metabolism | 7.80E-06 | 5.108 | 3.12E-04 | 2.99E-05 | 0 |
| Histidine metabolism | 7.87E-06 | 5.1041 | 3.12E-04 | 2.99E-05 | 0.22131 |
| Butanoate metabolism | 8.14E-06 | 5.0895 | 3.12E-04 | 2.99E-05 | 0 |
| Glutathione metabolism | 8.21E-06 | 5.0856 | 3.12E-04 | 2.99E-05 | 0.36435 |
| Arginine biosynthesis | 8.29E-06 | 5.0813 | 3.12E-04 | 2.99E-05 | 0.11675 |
| Glyoxylate and dicarboxylate metabolism | 8.74E-06 | 5.0587 | 3.12E-04 | 2.99E-05 | 0.13757 |
| D-Glutamine and D-glutamate metabolism | 9.97E-06 | 5.0013 | 3.39E-04 | 2.99E-05 | 0.5 |
| Nitrogen metabolism | 9.97E-06 | 5.0013 | 3.39E-04 | 2.99E-05 | 0 |
| Aminoacyl-tRNA biosynthesis | 1.70E-05 | 4.7689 | 5.45E-04 | 4.72E-05 | 0 |
| Pantothenate and CoA biosynthesis | 1.77E-05 | 4.7521 | 5.49E-04 | 4.72E-05 | 0.00714 |
| beta-Alanine metabolism | 2.30E-05 | 4.6387 | 6.89E-04 | 5.81E-05 | 0 |
| Nicotinate and nicotinamide metabolism | 4.30E-05 | 4.3664 | 0.001247 | 1.03E-04 | 0.56711 |

^1^ P value of t’ test; ^2^ the P value of t’ test with Holm’ adjustment; ^3^ False discover rate.
