# Supplementary material for: Metabolic difference between patient-derived xenograft model of pancreatic ductal adenocarcinoma and corresponding primary tumor
Source: BMC Cancer. 2024 Apr 17;24:485. doi: 10.1186/s12885-024-12193-x (PMC11022326; doi:10.1186/s12885-024-12193-x)
Supplement: Supplementary file 7 — Supplementary Material 7 [file 12885_2024_12193_MOESM7_ESM.docx]

| **Table S7 The statistical result of metabolic pathway analysis of PDXG1 vs PDXG3** | | | | | |
| --- | --- | --- | --- | --- | --- |
| **Pathway Name** | **p** | **-log(p)** | **Holm p** | **FDR** | **Impact** |
| Pyruvate metabolism | 7.16E-07 | 6.15E+00 | 3.44E-05 | 1.86E-05 | 0.29859 |
| Glycolysis / Gluconeogenesis | 7.74E-07 | 6.11E+00 | 3.64E-05 | 1.86E-05 | 0.13055 |
| Selenocompound metabolism | 5.45E-05 | 4.26E+00 | 2.51E-03 | 6.90E-04 | 0 |
| Fructose and mannose metabolism | 1.37E-04 | 3.86E+00 | 6.15E-03 | 6.90E-04 | 0 |
| Amino sugar and nucleotide sugar metabolism | 1.37E-04 | 3.86E+00 | 6.15E-03 | 6.90E-04 | 0.08314 |
| Galactose metabolism | 1.80E-04 | 3.74E+00 | 7.75E-03 | 6.90E-04 | 0.03152 |
| Alanine, aspartate and glutamate metabolism | 1.98E-04 | 3.70E+00 | 8.33E-03 | 6.90E-04 | 0.53686 |
| Arginine biosynthesis | 2.21E-04 | 3.66E+00 | 9.05E-03 | 6.90E-04 | 0.11675 |
| Glyoxylate and dicarboxylate metabolism | 2.22E-04 | 3.65E+00 | 9.05E-03 | 6.90E-04 | 0.13757 |
| Arginine and proline metabolism | 2.23E-04 | 3.65E+00 | 9.05E-03 | 6.90E-04 | 0.12158 |
| D-Glutamine and D-glutamate metabolism | 2.23E-04 | 3.65E+00 | 9.05E-03 | 6.90E-04 | 0.5 |
| Nitrogen metabolism | 2.23E-04 | 3.65E+00 | 9.05E-03 | 6.90E-04 | 0 |
| Histidine metabolism | 2.27E-04 | 3.64E+00 | 9.05E-03 | 6.90E-04 | 0.22131 |
| Butanoate metabolism | 2.29E-04 | 3.64E+00 | 9.05E-03 | 6.90E-04 | 0 |
| Glutathione metabolism | 2.30E-04 | 3.64E+00 | 9.05E-03 | 6.90E-04 | 0.36435 |
| Porphyrin and chlorophyll metabolism | 2.30E-04 | 3.64E+00 | 9.05E-03 | 6.90E-04 | 0 |
| Aminoacyl-tRNA biosynthesis | 3.62E-04 | 3.44E+00 | 1.16E-02 | 1.02E-03 | 0 |
| Citrate cycle (TCA cycle) | 4.30E-04 | 3.37E+00 | 1.33E-02 | 1.15E-03 | 0.24338 |
| Nicotinate and nicotinamide metabolism | 6.30E-04 | 3.20E+00 | 1.89E-02 | 1.59E-03 | 0.42895 |
| beta-Alanine metabolism | 8.44E-04 | 3.07E+00 | 2.45E-02 | 2.02E-03 | 0 |
